# Fluorescent and Magnetic Radical Dendrimers as Potential Bimodal Imaging Probes

**DOI:** 10.3390/pharmaceutics15061776

**Published:** 2023-06-20

**Authors:** Songbai Zhang, Vega Lloveras, Yufei Wu, Juan Tolosa, Joaquín C. García-Martínez, José Vidal-Gancedo

**Affiliations:** 1 Institut de Ciència de Materials de Barcelona (ICMAB-CSIC), Campus UAB, 08193 Bellaterra, Spain; szhang@icmab.esywu@icmab.es (Y.W.); 2 State Key Laboratory of Biotherapy and Cancer Center, West China Hospital, Sichuan University, Chengdu 610041, China; 3 CIBER de Bioingeniería, Biomateriales y Nanomedicina, Instituto de Salud Carlos III, Campus UAB, 08193 Bellaterra, Spain; 4 Departamento de Química Inorgánica, Orgánica y Bioquímica, Facultad de Farmacia, Universidad de Castilla-La Mancha, C/José María Sánchez Ibáñez s/n, 02008 Albacete, Spain; juan.tolosa@uclm.es (J.T.); joaquinc.garcia@uclm.es (J.C.G.-M.); 5 Regional Center for Biomedical Research (CRIB), Universidad de Castilla-La Mancha, C/Almansa 13, 02008 Albacete, Spain

**Keywords:** organic radicals, dendrimers, radical dendrimers, fluorescence, quenching, imaging techniques, magnetic resonance imaging (MRI), optical fluorescence imaging (OFI)

## Abstract

Dual or multimodal imaging probes have emerged as powerful tools that improve detection sensitivity and accuracy in disease diagnosis by imaging techniques. Two imaging techniques that are complementary and do not use ionizing radiation are magnetic resonance imaging (MRI) and optical fluorescence imaging (OFI). Herein, we prepared metal-free organic species based on dendrimers with magnetic and fluorescent properties as proof-of-concept of bimodal probes for potential MRI and OFI applications. We used oligo(styryl)benzene (OSB) dendrimers core that are fluorescent on their own, and TEMPO organic radicals anchored on their surfaces, as the magnetic component. In this way, we synthesized six radical dendrimers and characterized them by FT-IR, ^1^H NMR, UV-Vis, MALDI-TOF, SEC, EPR, fluorimetry, and in vitro MRI. Importantly, it was demonstrated that the new dendrimers present two properties: on one hand, they are paramagnetic and show the ability to generate contrast by MRI in vitro, and, on the other hand, they also show fluoresce emission. This is a remarkable result since it is one of the very few cases of macromolecules with bimodal magnetic and fluorescent properties using organic radicals as the magnetic probe.

## 1. Introduction

Imaging techniques are decisive in the diagnosis and follow-up of many diseases and have become essential in clinical practice. Dual or multimodal imaging probes have emerged as potent tools that improve accuracy and detection sensitivity in illness diagnosis and treatment. Multimodal imaging probes are designed to overcome the inherent disadvantages of each imaging modality and take advantage of the complementary information they provide. A probe which incorporates dual or multiple imaging properties is preferred to using a mixture of various contrast agents.

Among the different imaging techniques, one of the most versatile and used is magnetic resonance imaging (MRI) due to its high spatial resolution, non-ionizing character, and unlimited depth penetration. Nowadays, the most widely used contrast agents (CAs) to induce significantly improved and enhanced tissue contrast in such a technique are those based on Gd(III) chelates. However, they present toxicity concerns, and alternative imaging probes are highly required [1,2].

Our group has been developing organic radicals for different applications for a long time [3,4,5,6,7,8] and, lately, we have been working with radical dendrimers, i.e., dendrimers fully functionalized by organic radicals, as an alternative to Gd(III) chelates [9,10,11,12,13,14,15,16,17,18,19]. Since they are formed by organic radicals, radical dendrimers present paramagnetic properties such as Gd-based CAs and can act like them mainly decreasing the longitudinal relaxation time (T_1_) of the water protons and improving the contrast of the image but minimizing concerns about toxic metal accumulation. In fact, organic radicals such as nitroxides have been shown to be nontoxic in vivo [20,21]. Anchoring many organic radicals to a dendrimer scaffold has resulted in a successful strategy since we obtained systems with high relaxivity and with radicals protected from bioreduction. In this way, the two main limitations of isolated organic radicals were overcome, i.e., their low relaxivity and rapid bioreduction. In addition, we demonstrated that radical dendrimers are not toxic either in vitro or in vivo [13,15,18].

On the other hand, another imaging technique with a promising future is optical fluorescence imaging (OFI). Optical fluorescence imaging also has a non-ionizing character and it is commonly used in histologic analysis of cells, to monitor biodistribution, and has drawn interest from the medical community since it has potential for intraoperative use such as in imaging-guided surgery [22]. OFI has the main advantage of high sensitivity compared with MRI but it is limited by depth penetration because biological tissue attenuates light and scatters photons, and presents lower spatial resolution in vivo [23]. High spatial resolution and unrestricted depth penetration are advantages of MRI and low sensitivity is its drawback. By combining these two imaging techniques, the imaging result can be substantially improved.

In this work, we aimed to prepare metal-free organic species based on dendrimers not only with magnetic properties but also with fluorescent ones, for potential MRI and optical fluorescence imaging applications.

MRI/OFI imaging probes with dual modalities have been reported in some literature sources, using, the majority of them, Gd(III) chelates as the magnetic component (although the use of Fe_3_O_4_ or ^19^F, among others, has also been reported) and different fluorophores [24,25,26]. For example, a bimodal fluorescence-magnetic resonance probe was synthesized based on DOTA-Gd(III) chelate and tetraphenylethylene as aggregation-induced emission luminogen (AIEgen), for apoptosis imaging [27].

However, very few examples have been reported on bimodal MRI/OFI imaging probes using organic radicals as the MRI probe. In fact, this is a challenging goal since, in general, when a nitroxyl radical is close to a fluorophore, the fluorescence is quenched. Different mechanisms have been proposed to explain this phenomenon such as exchange-induced relaxation processes [28], intersystem crossing [29], energy transfer interactions [30], or electron transfer [31]. These very few reported cases use polymers as scaffolds [32,33,34]. Rajca and Johnson’s group prepared bimodal MRI and fluorescent contrast agents based on organic radicals (nitroxides) and Cy5.5 fluorophores, although both agents could not work at the same time. On branched-bottlebrush polymers, the fluorescence was quenched by the radicals but once the nitroxides were reduced by ascorbate, the fluorescence intensity increased by 2 to 3.5 times [33]. The same group prepared polymeric nanoparticles with the same active agents but in that case, it could be achieved simultaneously MRI and NIRF imaging in vivo due to the larger distance between the fluorophore and the nitroxides [34].

**Figure 1:**
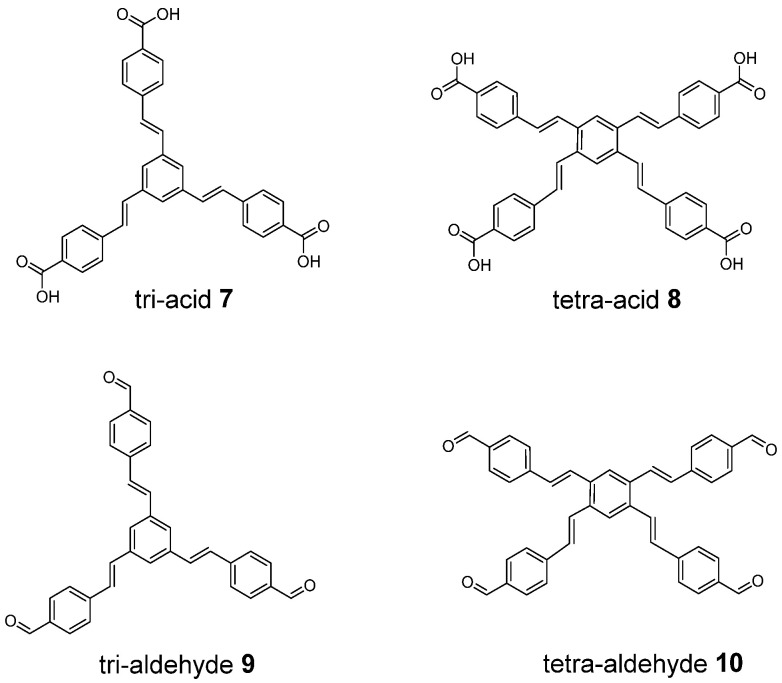
Chemical structures of oligo(styryl)benzene dendrimers cores (tri-acid **7**, tetra-acid **8**, tri-aldehyde **9,** and tetra-aldehyde **10**) derivatives used in this work to synthesize radical dendrimers.

Some of the ways used to impart fluorescence to dendrimers are by anchoring or encapsulating fluorescent dyes in the dendrimer’s structure. However, in this work, we used a different strategy, that is, the use of dendrimers that are fluorescent by themselves. With this strategy, additional synthetic steps due to the anchoring of fluorophores can be avoided, and it is, therefore, a less time-consuming method. In addition, at the same time, in this way all the end groups of the branches are free for full functionalization with organic radicals, resulting in a more optimal strategy. We used oligo(styryl)benzene (OSB) dendrimers cores (Figure 1) that present fluorescent properties on their own and are biocompatible [35]. We synthesized new radical dendrimers based on them, exploring their magnetic and fluorescent properties as proof of concept for potential MRI and OFI applications.

## 2. Materials and Methods

### 2.1. Materials

All reactants were purchased from Sigma Aldrich Inc. (St. Louis, MO, USA) at the highest purity available and used without further purification. THF, CH_2_Cl_2_, DMSO, and ethanol (HPLC grade) were obtained from SDS-Carlo Erba (Sabadell, Spain). CH_2_Cl_2_ was distilled over CaH_2_ and THF over metallic sodium.

### 2.2. Methods

**Chromatography**. Thin Layer Chromatography (TLC) was performed on Merck 60F254 silica plates and were visualized by UV (254 nm), or by ninhydrin. Silica column chromatography was carried out using silica gel 60 (35–70 mesh).

**Size exclusion chromatography (SEC)** analysis was carried out using an Agilent 1260 infinity II liquid chromatography system apparatus equipped with a diode array detector under the following conditions: a PSS SDV pre-column (3 μm, 8 × 50 mm) and a PSS SDV analytical column (3 μm, 1000 Å, 8 × 300 mm) with a diode array detector were used. CHCl_3_ was used as an eluent at a flow rate of 0.5 mL/min at 35 °C. Radical dendrimers were dissolved in the eluent to reach a final concentration of 1 mg/mL and filtered through a 0.2 μm PTFE filter before injection.

**Nuclear Magnetic Resonance spectroscopy (NMR)** ^1^H NMR (250 MHz) spectroscopy was performed at Bruker spectrometer Avance DRX-250. Chemical shifts (δ) are expressed in parts per million downfield from tetramethylsilyl chloride. The following abbreviations are used to indicate multiplicity: s: singlet; d: doublet, m: multiplet.

**Mass spectrometry**. Matrix-assisted laser desorption/ionization-time-of-flight mass (MALDI-TOF) BIFLEX spectrometer (Bruker-Franzen Analytik) equipped with a pulsed nitrogen laser (337 nm), using 19 kV acceleration voltage, at UAB. Dithranol was used as a matrix.

**Electron Paramagnetic Resonance spectroscopy (EPR)** spectra were obtained with an X-Band (9.7 GHz) Bruker ELEXSYS E500 spectrometer equipped with an ST8911 microwave cavity, a Bruker variable temperature unit, a field frequency lock system Bruker ER 033 M and an NMR Gaussmeter Bruker ER 035 M. The modulation amplitude was kept well below the line width, and the microwave power was well below saturation. Samples were previously degassed with Ar.

**Fourier-transform infrared spectroscopy (FT-IR)** spectra were recorded in a FT/IR-4700 spectrophotometer from JASCO (Tokyo, Japan) with an ATR (attenuated total reflectance) accessory, in the 400–4000 cm^−1^ range with 4 cm^−1^ resolution.

**Ultraviolet-visible spectroscopy (UV-Vis)** spectra were recorded in a UV-Vis-Nir JASCO V-780 spectrophotometer (Tokyo, Japan), in the 200–800 nm range.

**Fluorescence spectroscopy** emission spectra were recorded in a Varian Cary Eclipse fluorimeter from the Laboratori de Luminiscència i Espectroscòpia de Biomolècules (LLEB), at the UAB, in the range 320–800 nm, excitation slit 5 nm, emission 5 nm.

**Magnetic Resonance Imaging (MRI)** experiments were carried out in a BioSpec 70/30 Bruker system using a 7.0 T horizontal-bore superconducting magnet equipped with actively shielded gradients (B-GA12 gradient coil inserted into a B-GA20S gradient system). A quadrature 72 mm inner diameter volume coil was used for in vitro studies.

**Relaxometric measurements**. Longitudinal (r_1_) relaxivities were determined per concentration of (2,2,6,6-Tetramethylpiperidin-1-yl)oxyl (TEMPO) units. Different concentrations of TEMPO radicals were prepared, from 10 to 0.63 mM. Relaxivity measurements were obtained at room temperature. The software used for the calculations of T_1_ relaxations was Paravision 6.0 (Bruker Software). **r_1_ relaxivity**. T_1_ maps were performed with Rapid Acquisition with Relaxation Enhancement (RARE) sequence with a variable repetition time (VTR). Series of axial T_1_-weighted (T_1_W) images were acquired for each concentration of TEMPO to obtain T_1_ maps based on a magnetization saturation experiment and the following parameters: Images at 18 different TR values were acquired: TR = 50, 80, 120, 160, 200, 250, 300, 360, 420, 500, 650, 850, 1100, 1600, 2200, 3000, 5000, and 10,000 ms. T_1_ measurements were acquired with a reduced echo time of 6 ms to minimize signal loss due to T_2_. Field of view (FOV) = 2.5 × 2.5 cm, averages (Av) = 1, acquisition matrix (Mtx) = 128 × 128. The T_1_ values were calculated from the mean signal in the region of interest (ROI) for each repetition time, adjusted to the following equation: *S* = *S_o_* [1 − (−TR/T_1_)].

## 3. Results and Discussion

### 3.1. Synthesis and Characterization of Radical Dendrimers Based on Oligo(styryl)Benzenes

We first synthesized oligo(styryl)benzene dendrimers with three and four branches ended in carboxylic acid and aldehyde groups, as previously reported [35,36], named in this work tri-acid (**7**), tetra-acid (**8**), tri-aldehyde (**9**) and tetra-aldehyde (**10**) (Figure 1). Briefly, the synthesis is based on the Horner-Wadsworth-Emmons reaction for the formation of the double bonds in situ from phosphonate tri- and tetra-substituted benzenes and the corresponding precursor aldehyde. These compounds are considered the core of a family of dendrimers.

Then, we synthesized a series of radical dendrimers based on them by coupling the amino group of 4-amino-TEMPO radical to the carboxyl or aldehyde end groups, leading to amido- and imino-radical dendrimers derivatives, respectively. In addition, the imino-derivatives were reduced to the corresponding amino-derivatives since a secondary amine is a more stable linker (see the synthesis details in the Supplementary Materials). We are using these compounds as a proof of concept for the future higher generations of radical dendrimers that we plan to develop using the same fluorescent core.

**Scheme 1:**
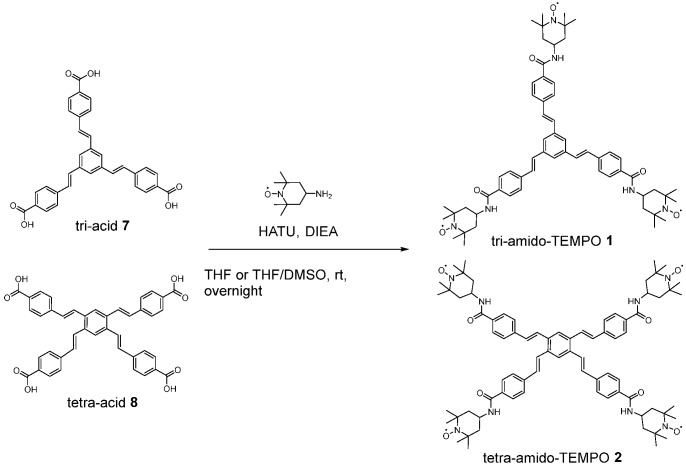
Synthesis of tri-amido-TEMPO **1** and tetra-amido-TEMPO **2** radical dendrimers.

**Scheme 2:**
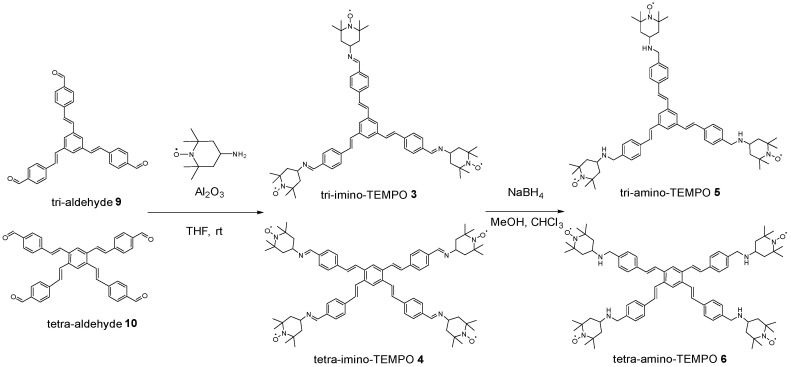
Synthesis of tri-imino-TEMPO **3**, tetra-imino-TEMPO **4**, tri-amino-TEMPO **5,** and tetra-amino-TEMPO **6** radical dendrimers.

In this way, we obtained six radical dendrimers (**1**–**6**, Scheme 1 and Scheme 2) which were characterized by FT-IR, ^1^H NMR, UV-Vis, MALDI-TOF, SEC, and EPR. Moreover, fluorescence and MRI studies were carried out to check whether bimodality has been achieved in these species or not.

**Figure 2:**
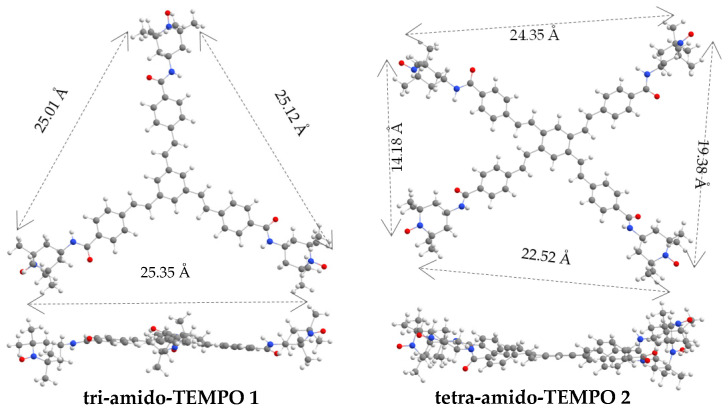
Optimized geometric structures of radical dendrimers **1**–**2** at UB3LYP-D3(BJ)/6–31G(d) level in the gas phase. See Scheme S1 for the corresponding optimized geometric structures of compounds **3**–**6**.

Finally, we also optimized the geometry structure of the obtained radical species **1**–**6** at UB3LYP-D3(BJ)/6–31G(d) level in the gas phase using Gaussian 16 (see Figure 2 for **1**–**2** structures and Scheme S1 for **3**–**6** ones). The optimized structures are similar to those of their dendrimers precursors [37]. They are constituted by a highly conjugated backbone of π-bonds that confers a high rigidity. In the case of the tri-substituted compounds, the styrylbenzene branches are arranged towards the apexes of a triangle while the tetra-substituted compounds present a cross conformation of the styrylbenzene branches with a higher conjugation than the three-styrylbenzene system [37]. The incorporation of TEMPO radicals at the dendrimers’ surface through a flexible functionalization does not affect the conjugated backbone so their arrangement is fixed and determined by the rigid separation of the structure. The distances between the N-O^•^ groups of the radicals in the tri-substituted compounds are larger (ca. 25 Å) than in the four-substituted analogs in which the shortest distance is between 11 and 14 Å.

#### 3.1.1. Synthesis and Characterization of Amido Radical Dendrimers Derivatives **1** and **2**

The coupling of 4-amino-TEMPO to the carboxyl end groups of OSB tri-acid **7** and tetra-acid **8** has been carried out using HATU/DIEA as a coupling agent, as shown in Scheme 1. The synthesis of tri-amido-TEMPO **1** was performed in THF, while for tetra-amido-TEMPO **2**, a small amount of DMSO was added to make tetra-acid **8** soluble in the reaction system. After an overnight reaction at room temperature, the products were first extracted in dichloromethane/water and then purified by column chromatography on silica gel in dichloromethane/EtOH. The obtained yields were 88 and 53%, for tri-amido-TEMPO **1** and tetra-amido-TEMPO **2**, respectively (see the synthesis details of **1**–**2** compounds in the Supplementary Materials).

The IR spectra of tri-acid dendrimer (**7**) and the corresponding tri- and tetra-amido-TEMPO (**1** and **2**) radical dendrimers are shown in Figure S1. We followed up the reaction by IR, by the shift of the C=O stretching band from 1674 cm^−1^ (-COOH group) to 1632 cm^−1^ (-CONH amido group) for both compounds, as well as by the disappearance of the very broad OH stretching band at 3250–2500 cm^−1^ from the -COOH groups, and the appearance of the new NH stretching band from the amido group at 3299 and 3297 cm^−1^ for tri- and tetra-amido-TEMPO, respectively. In addition, we can observe the -CH stretching bands of -CH_3_ and -CH_2_- groups of TEMPO radicals at the range ca. 2854–2971 cm^−1^, and the bands assigned to the N-O^•^ stretching of the TEMPO radicals at 1362–64 cm^−1^ [38,39].

**Figure 3:**
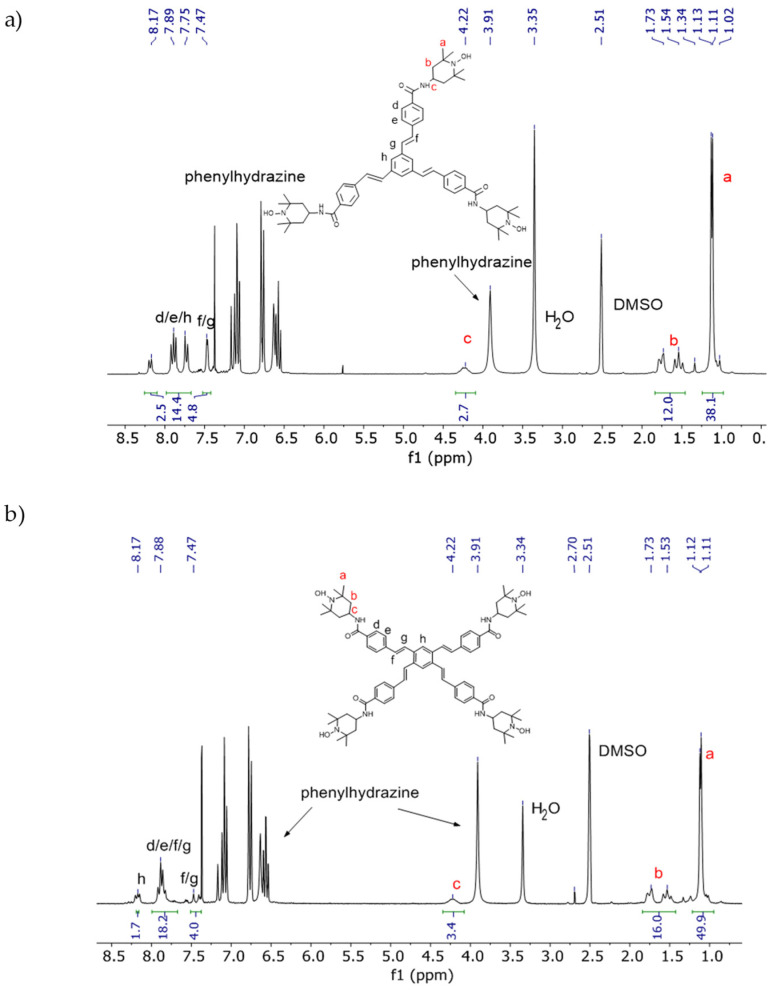
^1^H NMR spectra of (**a**) tri-amido-TEMPO (**1**) and (**b**) tetra-amido-TEMPO (**2**) after being reduced with phenylhydrazine (DMSO-d_6_, 250 MHz).

In order to characterize by ^1^H NMR such species, we used phenylhydrazine to reduce the nitroxyl radicals to the corresponding diamagnetic hydroxylamine (Scheme S2). In Figure 3, we can observe both ^1^H NMR spectra with the corresponding peak labeling. The peaks of the TEMPO protons can be found in the range of 1–1.73 ppm (*a* and *b*) and at 4.22 ppm (*c*) while the protons of the dendrimer structure (*d*, *e*, *f*, *g*, *h*) can be found between 7.47 and 8.17 ppm. Moreover, the relative integral values of the ^1^H resonances were in agreement with the theoretical ones, confirming the number of TEMPO radicals anchored on their structures (3 and 4, respectively).

Tri- and tetra-amido-TEMPO (**1** and **2**) were also characterized by UV-Vis spectroscopy (see Figures S2–S4 of the Supplementary Materials). By UV-Vis spectroscopy, we can quantify the number of TEMPO radical units anchored to the dendrimers since the low-intensity n-π* transition band at ca. 450 nm from the TEMPO radical is known to be additive. The molar extinction coefficient (*ε*) of the ca. 450 nm band of TEMPO radical is around 10 M^−1^ cm^−1^, with small variations depending on the solvent used [17]. The corresponding *ε* value for tri-amido-TEMPO (**1**) in dichloromethane was 30.6 M^−1^ cm^−1^, indicating 3 times higher molar extinction coefficient than the free TEMPO in the same solvent (11.1 M^−1^ cm^−1^, Figure S2) confirming that three radicals were coupled to the dendrimer. Unfortunately, it was not possible to confirm by UV-Vis spectroscopy the degree of substitution in the tetra-amido-TEMPO (**2**) since the higher conjugation of the tetra-styrylbenzene dendrimer, compared with the tri-substituted one, resulted in a red shift of the absorption bands which overlapped with the TEMPO absorption band (Figure S4).

By MALDI-TOF mass spectrometry, we observed the molecular ion peaks [M + H]^+^ of tri- and tetra-amido-TEMPO **1** and **2** at *m*/*z* = 977.2 and 1277.2, respectively, which are in agreement with the theoretical molecular mass (976.30 and 1275.69 Da, respectively). Also, it was observed a [M + Na]^+^ cluster in both cases (Figure S5).

**Figure 4:**
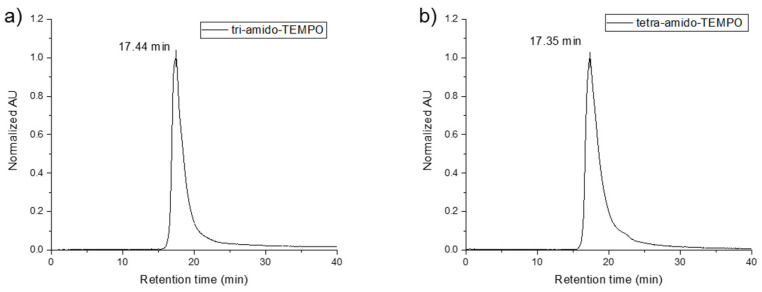
SEC chromatograms of (**a**) tri-amido-TEMPO (**1**) and (**b**) tetra-amido-TEMPO (**2**) radical dendrimers.

SEC was used to check the purity of both tri- and tetra-amido-TEMPO **1** and **2**. Both compounds showed only one narrow size distribution band. The slightly lower retention time obtained for tetra-amido-TEMPO **2** was in agreement with the slightly larger size than tri-amido-TEMPO **1** (Figure 4).

#### 3.1.2. Synthesis and Characterization of Imino and Amino Radical Dendrimers Derivatives (**3**–**6**)

To obtain the imino derivative compounds, tri- and tetra-aldehyde dendrimers (**9** and **10**) were subjected to sonification in the presence of alumina gel with an excess of 4-amino-TEMPO in THF [40,41] (Scheme 2). The reaction completion was also followed up by IR, by the disappearance of the aldehyde band. The alumina gel was removed by filtration, and the resulting product precipitated from the reaction mixture by the addition of *n*-pentane. In this way, we obtained the radical dendrimers tri-imino-TEMPO (**3**) and tetra-imino-TEMPO (**4**) in 63% and 85% yields, respectively.

On the other hand, the synthesis of tri- and tetra-amino-TEMPO (**5** and **6**) derivatives was performed by reduction of tri- and tetra-imino-TEMPO (**3** and **4**) with NaBH_4_, as shown in Scheme 2. After their reduction with NaBH_4_ overnight in methanol and chloroform, the reaction mixture was extracted with dichloromethane/water three times, and the products were obtained in 97% and 95% yields, respectively. See the synthesis details of **3**–**6** compounds in the Supplementary Materials.

As well as in compounds **1** and **2**, radical dendrimers **3**–**6** were also successfully characterized by IR, ^1^H NMR, UV-Vis, MALDI-TOF, and SEC, confirming their structure and purity. See the Supplementary Materials for the full characterization of **3**–**6** with these techniques (Figures S6–S16).

#### 3.1.3. EPR Study of the Radical Dendrimers **1**–**6**

**Figure 5:**
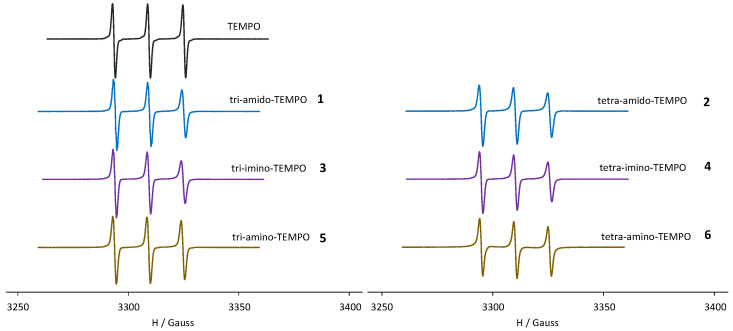
EPR normalized spectra of TEMPO, tri-amido-TEMPO **1**, tetra-amido-TEMPO **2**, tri-imino-TEMPO **3**, tetra-imino-TEMPO **4**, tri-amino-TEMPO **5** and tetra-amino-TEMPO **6** radical dendrimers in THF at 300 K.

The EPR spectrum of TEMPO free radical at 300 K showed the typical 3-line spectrum of nitroxides, with the same relative intensities 1:1:1, a coupling constant with the ^14^N atom *a*_N_ = 15.7 G, a line width of ΔHpp = 1.20 G and a *g*-factor = 2.0061 (Figure 5). The EPR spectra of the radical dendrimers **1**–**6** at 300 K also showed 3 lines, indicating that they showed negligible spin exchange interaction between their radical units, probably because of the rigidity of the structures that do not allow radicals to approach each other. They presented similar *a*_N_ and *g* factor than the TEMPO free radical (*a*_N_ ~ 15.4/15.5 G and *g* between 2.0053 and 2.0064, Table 1). However, the line width of their spectrum lines was slightly broader (~1.60 G) and they presented a selective decrease in the high-field line. These two features are due to the impeded motion of the radicals when they are attached to a large molecule, confirming, thus, the anchoring of the radicals to the dendrimers.

On the other hand, the EPR signal intensity (the area from the double integral) of the tri- and tetra-compound derivatives was found to be around three and four times higher than the TEMPO free radical (see Table 1).

In frozen solution, 120 K (Figure S17), the EPR spectra shape of the radical dendrimer species with three radical units was similar to the obtained with the TEMPO free radical, thus, showing weak dipole-dipole interactions among the TEMPO radicals. In fact, the empirical ratio *d*_1_/*d* values, which indicate the strength of the dipolar interactions, were only slightly higher than for TEMPO free radical (see Table 1). However, in tetra-substituted derivatives, these interactions were a bit larger, which means radicals are closer in those structures, in agreement with the distances obtained from the optimized structures (Figure 2), i.e., shorter radical–radical distance in the four-substituted derivatives than in the three-substituted ones.

Under these conditions, all compounds showed a |Δ*m*_s_| = 2 transition at half-field (Figure S18), typical of dipolar coupled spins that demonstrates a high-spin state is present. This signal was more intense in compounds with four radical units than in the tri-substituted analogs.

### 3.2. Fluorimetry Study of the OSB Precursors **7**–**10** and Radical Dendrimers **1**–**6**

In order to check the effect of nitroxyl radicals on the fluorescent properties of the OSB derivatives, we measured the fluorescence intensity of OSB precursors **7**–**10** and the corresponding radical dendrimers **1**–**6** in THF, using quinine sulfate as standard.

**Figure 6:**
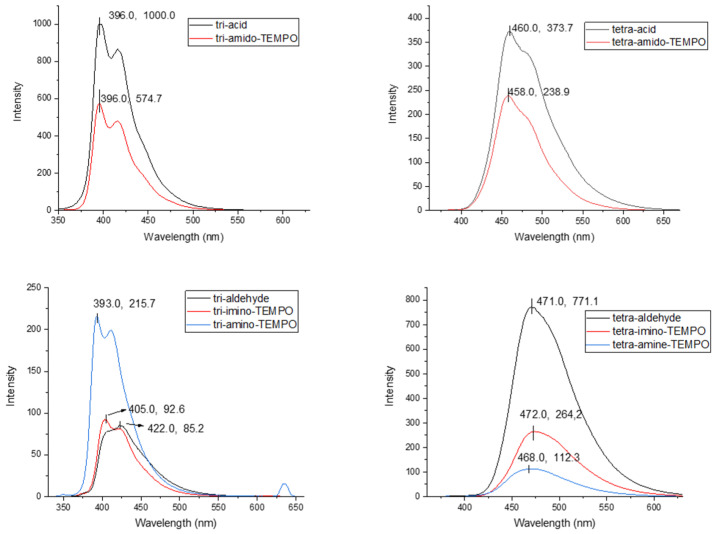
Fluorescence emission spectra of OSB precursors **7**–**10** and amido-, imino- and amino-radical dendrimers derivatives **1**–**6**, in THF.

The corresponding UV-Vis spectra and fluorescence emission spectra are shown in Figure 6 and Figure S19, respectively, while the calculated quantum yields (QY), λ_exc._ and λ_em._ values are shown in Table 2. See more details about the fluorescence process and the quantum yield calculation method in the Supplementary Materials.

According to the spectroscopic results obtained it is observed that the absorption occurs mainly on the part of the oligo(styryl)benzene units since both the absorbance maximum and the shape of the bands between the precursor dendrimers and the radical dendrimers are coincident. The optical properties of OSBs are highly dependent on their peripheral substituents [42,43]. Specifically, for the tri-acid **7** and tri-aldehyde **9** compounds, a previous study indicates that these absorptions correspond mainly to various contributions from S_0_→S_1_ and S_0_→S_2_ transitions and the attached functional groups participate in these transitions by stabilizing or destabilizing the HOMO or LUMO [44]. The tetra-substituted derivatives show more red-shifted absorbances. When comparing the tri-amido-TEMPO **1** and tetra-amido-TEMPO **2** derivatives with the starting precursors (tri-acid **7** and tetra-acid **8**, respectively), it is observed that there is no substantial variation in the absorbance maximum for each of them. This is because the electronic characteristics of the OSB do not change substantially since the differences between the electronic properties of the acid group and the amido group are not large enough to modify the OSB since both are electron-withdrawing groups. However, when comparing the imino- and amino-radical dendrimers (**3**–**6**) with the tri- and tetra-aldehyde precursors (**9**–**10**), it is observed that the formation of the imine does not produce a change in the electronics of the OSB but its reduction to form the amino. The reduction transforms an sp^2^ carbon of the imine into an sp^3^ carbon which interrupts the communication between the amino group and the OSB. This results in a hypsochromic shift (or blue shift) of the absorption band. The electron attracting groups such as aldehyde or imine remove electron density from the HOMO, destabilizing it, increasing its energy, and reducing the gap between HOMO and LUMO, leading to absorption bands at 340 nm for tri-substituted and at 360 nm for tetra-substituted. However, when this interaction between the functional group and the OSB is broken (due to the amine group formation), the HOMO stabilizes (i.e., the HOMO-LUMO gap increases), and the observed absorption is slowed blue shifted (319 nm for the tri-amino-TEMPO **5** and 344 nm for the tetra-amino-TEMPO **6**).

This effect is less important at the emission wavelength, where the fluorescence of each radical dendrimer is very similar to that of the precursor OSB. This indicates that the excited state from which the emission starts is very similar in both the radical dendrimer and the precursor and is mainly centered on the OSB backbone. Previous work on OSB indicates that tri-substituted acidic compounds exhibit better quantum yields than their four-branched analogs. This is because increasing the number of stilbene units increases the vibrational and rotational motions in a so-called restriction of intramolecular motions (RIM) mechanism [45], all responsible for the non-radiative relaxations of the absorbed light from the molecule. In the case of aldehyde derivatives, the 3-branched compound presents worse quantum yields because it undergoes a cis/trans photoisomerization process that is not possible in the 4-branched compound due to steric hindrance. It is important to remark that all the radical dendrimers synthesized show fluorescence, although the incorporation of radicals into OSB scaffolds leads, in most cases, to a decrease in fluorescence intensity and hence in the quantum yield. In any case, they are still acceptable quantum yields, in general (between 20.1% and 4.0%, Table 2). In tri-amido-TEMPO **1** and tetra-amido-TEMPO **2**, the quantum yield decreases to half, approximately, with respect to tri-acid **7** and tetra-acid **8**, respectively. In the case of tetra-imino-TEMPO **4** and tetra-amino-TEMPO **6**, their fluorescence quantum yield is quenched with respect to tetra-aldehyde **10** dendrimer until also approximately half in the former and one-fifth, in the latter. On the other hand, the quantum yield of tri-imino-TEMPO **3**, around 4% (0.04), is similar to its corresponding precursor tri-aldehyde **9** dendrimers (0.041), i.e., in this case, the anchoring of radicals does not quench the fluorescence. However, the quantum yield of tri-amino-TEMPO **5** is double (0.073) from that of the tri-aldehyde **9** dendrimer (0.041). In this case, the fluorescence values are very low, both in the precursor molecule and in the radical dendrimer, so any changes may appear to greatly affect the quantum yields. It is true that it has been described how amines not directly bound to chromophore systems can give rise to special fluorescence phenomena called the photoinduced electron transfer (PET) effect and that cannot be ruled out in this system. In any case, the radical dendrimers studied showed good fluorescent properties that allow them to be used as fluorescent probes [36].

### 3.3. MRI Measurements

The ability to generate contrast, i.e., the ability to decrease the T_1_ relaxation time of the protons of the solvent, has been evaluated in vitro for tri-amido-TEMPO **1**, tri-imino-TEMPO **3**, tri-amino-TEMPO **5** and tetra-amido-TEMPO **2** derivatives, as well as for TEMPO radical, used as a reference, in organic solvents.

Tri-amido-TEMPO **1**, tri-imino-TEMPO **3,** and tri-amino-TEMPO **5** derivatives were studied in dichloromethane (Figure S20) and their relaxivities *r*_1_ are listed in Table 3. The three compounds present similar *r*_1_ relaxivity values ranging from 0.316 to 0.339 mM^−1^ s^−1^. Interestingly, we can observe in Table 3 that their corresponding relaxivity value per radical unit (ca. 0.11 mM^−1^ s^−1^) is higher than that for the free TEMPO (0.080, around 30% higher). This likely results from the larger size of dendrimers and their high rigidity [46].

**Figure 7:**
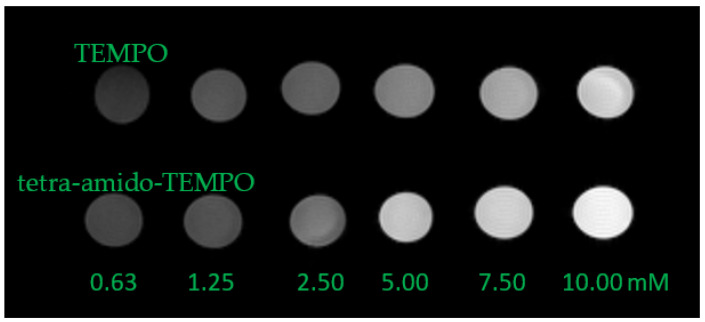
Phantoms T_1_-weighted images of TEMPO radical and tetra-amido-TEMPO **2**, at the same concentrations per unit of TEMPO radical in both cases (in green), in DMSO.

In addition, the relaxivity values of tri-amido-TEMPO **1** and tetra-amido-TEMPO **2** radical dendrimers were also obtained, in DMSO, as well as for the free TEMPO for comparison (Figure 7 and Figure S21 and Table 4). The *r*_1_ relaxivity values of tri-amido-TEMPO **1** and tetra-amido-TEMPO **2** are 0.530 and 0.828 mM^−1^ s^−1^, respectively, i.e., higher in the 4-substituted compound, as expected. Taking into account the corresponding values per radical unit, they were significantly higher than that for the free TEMPO (Table 4). Similarly to the results obtained for the tri-substituted compounds, the dendrimer with three TEMPO units showed an increase of the relaxivity per radical unit of around 30% and, remarkably, the dendrimer with four TEMPO units showed an increase of the relaxivity per radical unit of around 50%. These results confirmed that the higher molecular size and rigidity seem beneficial to increase the relaxivity.

## 4. Conclusions

We synthesized six radical dendrimers based on fluorescent oligo(styryl)benzenes, with different linking bonds between the dendrimer branches and the radicals (amido, imino, and amino linkers). They were fully characterized by IR, ^1^H NMR, UV-Vis, MALDI-TOF, SEC and EPR confirming that the dendrimers were fully substituted with TEMPO radicals.

Remarkably, all the synthesized radical dendrimers maintain fluorescent properties after the radical coupling, although the fluorescence quantum yield is decreased in almost all cases with respect to their corresponding OSB precursors. In addition, in vitro MRI studies of tri-amido-TEMPO (**1**), tri-imino-TEMPO (**3**), tri-amino-TEMPO (**5**), and tetra-amido-TEMPO (**2**) have shown that they have the capacity to decrease the T_1_ relaxation time of the solvent protons, and, also, they show a relaxivity *r*_1_ per unit of radical higher than the TEMPO free radical. Thus, we obtained bimodal fluorescent-magnetic species, as a proof of concept, for potential promising bimodal imaging applications. Tetra-amido-TEMPO (**2**) is one of the best candidates due to its good performance in both fluorescence and relaxivity.

## Figures and Tables

**Table 1:** EPR data of TEMPO and radical species **1**–**6** at 300 K and at 120 K, in THF.

Compound	300 K			120 K	
	*g*	*a*_N_ (G)	ΔHpp (G)	*d*_1_/*d*	Area (a.u.)
TEMPO	2.0061	15.7	1.20	0.53	2.47 × 10^5^
tri-amido-TEMPO (**1**)	2.0059	15.5	1.61	0.66	7.22 × 10^5^
tri-imino-TEMPO (**3**)	2.0060	15.4	1.59	0.63	7.12 × 10^5^
tri-amino-TEMPO (**5**)	2.0057	15.4	1.62	0.60	7.31 × 10^5^
tetra-amido-TEMPO (**2**)	2.0064	15.5	1.64	0.80	9.49 × 10^5^
tetra-imino-TEMPO (**4**)	2.0053	15.4	1.56	0.79	9.61 × 10^5^
tetra-amino-TEMPO (**6**)	2.0057	15.4	1.40	0.75	9.52 × 10^5^

**Table 2:** The calculated quantum yield of quinine sulfate, dendrimers **7**–**10,** and radical dendrimers **1**–**6**.

Compound	λ_exc_ (nm)	λ_em_ (nm)	Abs. (A)	FL Area (F)	Refraction Index (R)	Quantum Yield
quinine sulfate	348	452	0.074	65,192.32	1.33	0.540
tri-acid **7**	326.5	396	0.0899	52,371.05	1.4072	0.400
tri-amido-TEMPO **1**	327	396	0.0967	28,274.02	1.4072	0.201
tetra-acid **8**	350	460	0.0833	30,086.73	1.4072	0.248
tetra-amido-TEMPO **2**	338	458	0.0954	16,902.84	1.4072	0.122
tri-aldehyde **9**	340	422	0.0985	5922.78	1.4072	0.041
tri-imino-TEMPO **3**	338	405	0.0891	5171.24	1.4072	0.040
tri-amino-TEMPO **5**	319	393	0.0971	12,236.02	1.4072	0.086
tetra-aldehyde **10**	364	471	0.0961	59,755.09	1.4072	0.427
tetra-imino-TEMPO **4**	361	472	0.0801	21,039.81	1.4072	0.180
tetra-amino-TEMPO **6**	344	468	0.0859	9150.83	1.4072	0.073

**Table 3:** Relaxivity values (*r*_1_) for free TEMPO, tri-amido-TEMPO **1**, tri-imino-TEMPO **3,** and tri-imino-TEMPO **5**, in dichloromethane.

Compound	*r*_1_ (mM^−1^ s^−1^) per Molecule	*r*_1_ (mM^−1^ s^−1^) per Nitroxide Unit
TEMPO	0.079	0.079
tri-amido-TEMPO **1**	0.316	0.105
tri-imino-TEMPO **3**	0.339	0.113
tri-amino-TEMPO **5**	0.321	0.107

**Table 4:** Relaxivity values (*r*_1_) for free TEMPO, tri-amido-TEMPO (**1**) and tetra-amido-TEMPO (**2**), in DMSO.

Compound	*r*_1_ (mM^−1^ s^−1^) per Molecule	*r*_1_ (mM^−1^ s^−1^) per Radical Unit
TEMPO	0.139	0.139
tri-amido-TEMPO **1**	0.530	0.177
tetra-amido-TEMPO **2**	0.828	0.207

## Data Availability

Not applicable.

## Supplementary Materials

The following supporting information can be downloaded at: https://www.mdpi.com/article/10.3390/pharmaceutics15061776/s1, synthesis and characterization of compounds **1**–**6**; optimized geometric structures of compounds **3**–**6**, the rest of FT-IR, UV-Vis, ^1^H NMR, MALDI-TOF, SEC chromatograms and EPR spectra of **1**–**6**; UV-Vis spectra for the QY calculation of **1**–**6**, QY calculation method, Jablonski diagram and plots of *R*_1_ (1/T_1_) versus concentration of **1**, **2**, **3** and **5**.
