# Supplementary material for: Fluorescent and Magnetic Radical Dendrimers as Potential Bimodal Imaging Probes
Source: Pharmaceutics. 2023 Jun 20;15(6):1776. doi: 10.3390/pharmaceutics15061776 (PMC10300780; doi:10.3390/pharmaceutics15061776)
Supplement: Supplementary file 1 [file pharmaceutics-15-01776-s001.zip › pharmaceutics-2381986-supplementary.pdf]

Supplementary Materials: Fluorescent and magnetic radical dendrimers as potential bimodal imaging probes

Songbai Zhang [1], Vega Lloveras[1,*], Yufei Wu [1], Juan Tolosa, Joaquín C. García, and José Vidal-Gancedo[1,*]

1.Synthesis and characterization

*1.1. tri-amido-TEMPO (**1**)*

20.0 mg of tri-acid dendrimer **7** (0.13 mmol, 1 eq) were added into a round-bottom flask and dissolved in 6 mL of THF_{anh}, then 49.74 mg of HATU (0.13 mmol, 3.3 eq) were added under argon atmosphere. 22.4 mg of 4-amino-TEMPO (0.13 mmol, 3.3 eq) were dissolved in a vial with 1 mL of THF_{anh} and transferred into the flask containing the tri-acid and HATU. After the solution was stirred for 10 min, 22.7 μL of DIEA (0.13 mmol, 3.3 eq) were added into the flask with a syringe and the mixture was stirred overnight. Then, THF was removed under vacuum, and the solid was dissolved in dichloromethane (DCM). The organic phase in DCM was extracted five times with MiliQ water and dried with $MgSO_4$. After that, the product tri-amido-TEMPO (**1**) was obtained after purification by column chromatography on silica gel (DCM/EtOH, 40/1, v/v, Rf=0.33), and being dried under vacuum, (33.5 mg, 87.67 %).

<u>Characterization:</u>

- ^1H NMR (phenylhydrazine) (250 MHz, DMSO-d_6): δ = 1.1 (d, 36H, H_a), 1.5-1.7 (m, 12H, H_b), 4.2 (s, 3H, H_c), 7.5-7.9 (m, 21H, H_d, H_e, H_f, H_g, H_h) ppm.
- IR (cm^{-1}): 3299 ν(NH), 2972 ν(CH), 2928 ν(CH), 2852 ν(CH), 1630 ν(CO), 1537, 1500, 1458, 1364, 1323, 1263, 1242, 1179, 964, 859, 847.
- MS (MALDI-TOF, positive mode): 977.2 m/z $[M+H]^+$. Calculated: $C_{60}H_{75}N_6O_6{}^{3\bullet}$, 976.30 Da.
- EPR (THF): g = 2.0059; a_N = 15.5 G; ΔH_{pp} = 1.61 G.

*1.2. tetra-amido-TEMPO (**2**)*

The synthesis of the tetra-amido-TEMPO (**2**) was similar to the synthesis of tri-amido-TEMPO (**1**). 20 mg of tetra-acid **8** (0.03 mmol, 1 eq) and 50.49 mg HATU (0.13 mmol, 4.4 eq) were added into a round-bottom flask, and 8 mL of THF_{anh} were added into the flask. 22.74 mg of 4-amino-TEMPO (0.13 mmol, 4.4 eq) were weighed and dissolved in a vial with 2 mL of THF_{anh} and transferred into the flask. After the solution was stirred for 10 min, 23 μL of DIEA (0.13 mmol, 4.4 eq) were added. The reaction mixture was stirred overnight. 1.5 mL of DMSO was added into the flask. After being stirred for another 4 hours, THF was removed under vacuum and the residue part was dissolved in DCM. The solution in DCM was extracted with water five times. The organic phase was dried with $MgSO_4$ and purified by column chromatography on silica gel (DCM/MeOH, 20/1, v/v, Rf=0.44) to obtain the product (21.1 mg, 53%).

<u>Characterization:</u>

- ^1H NMR (phenylhydrazine) (250 MHz, DMSO-d_6): δ = 1.1 (d, 48H, H_a), 1.5-1.7 (m, 16H, H_b), 4.2 (s, 4H, H_c), 7.5-8.2 (m, 26H, H_d, H_e, H_f, H_g, H_h) ppm.
- IR (cm^{-1}): 3297 ν(NH), 2970 ν(CH), 2925 ν(CH), 2854 ν(CH), 1632 ν(CO), 1600, 1531, 1498, 1458, 1362, 1320, 1267, 1240, 1176, 957, 864, 759.
- MS (MALDI-TOF, positive mode): 1277.2 m/z $[M+H]^+$. Calculated: $C_{78}H_{98}N_8O_8{}^{4\bullet}$, 1275.69 Da.
- EPR (THF): g = 2.0064; a_N = 15.5 G; ΔH_{pp} = 1.64 G.

*1.3. tri-imino-TEMPO (**3**)*

20.0 mg of tri-aldehyde **9** (0.04 mmol, 1 eq) were added into the flask and then 2 mL of THF were added to dissolve it. 24.76 mg of 4-amino-TEMPO (0.14 mmol, 3.3 eq) were weighed and put into a vial, and THF_{anh} (1 mL) was used to dissolve the radical. The solution of 4-amino-TEMPO was transferred into the flask containing the tri-aldehyde. Al_2O_3 (32 mg) was added into the flask to accelerate the reaction and we sonicated the reaction mixture for 6 h. After filtration to remove Al_2O_3, the reaction solution was precipitated with *n*-pentane twice. The product was obtained as solid and dried under vacuum (25 mg, 62.5%).

<u>Characterization:</u>

- ^1H NMR (phenylhydrazine) (250 MHz, DMSO-d_6): δ = 1.1 (d, 36H, H_a), 1.6 (m, 12H, H_b), 3.7 (s, 3H, H_c), 7.4-7.8 (m, 21H, H_e, H_f, H_g, H_h, H_i), 8.5 (d, 3H, H_d) ppm.

- IR (cm^{-1}): 3027, 2973 ν(CH), 2928 ν(CH), 2863 ν(CH), 1637 ν(C=N), 1600, 1509, 1560, 1414, 1360, 1346, 1306, 1240, 1216, 1173, 1046, 960, 849, 806, 566.
- MS (MALDI-TOF, positive mode): 929.2 m/z $[M+H]^+$. Calculated: $C_{60}H_{75}N_6O_3{}^{3\bullet}$, 928.30 Da.
- EPR (THF): g = 2.0060; a_N = 15.4 G; ΔH_{pp} = 1.59 G.

*1.4. tetra-imino-TEMPO (**4**)*

The synthesis of the tetra-imino-TEMPO (**4**) was similar to the synthesis of tri-imino-TEMPO (**3**). 20 mg of tetra-aldehyde **10** (0.03 mmol, 1 eq) were added into a flask and dissolved in THF_{anh} (8 mL). 22.9 mg of 4-amino-TEMPO (0.13 mmol, 4 eq) were weighed and dissolved with THF (2 mL) in a vial. The solution was transferred into the flask with a syringe. Al_2O_3 (30 mg) was added to the solution and the reaction mixture was stirred for 8h under sonication. After filtration to remove Al_2O_3 and precipitation with *n*-pentane three times, the product was obtained and dried under vacuum (34.4 mg, 85%).

Characterization:

- ^1H NMR (phenylhydrazine) (250 MHz, DMSO-d_6): δ = 1.1 (d, 48H, H_a), 1.6 (m, 16H, H_b), 3.6 (s, 4H, H_c), 7.5-8.1 (m, 26H, H_e, H_f, H_g, H_h, H_i), 8.5 (s, 4H, H_d) ppm.
- IR (cm^{-1}): 3030, 2971 ν(CH), 2930 ν(CH), 2855 ν(CH), 1637 ν(C=N), 1601, 1560, 1508, 1464, 1414, 1360, 1343, 1302, 1240, 1221, 1172, 1105, 1048, 957, 901, 856, 809, 532.
- MS (MALDI-TOF, positive mode): 1213.2 m/z $[M+H]^+$. Calculated: $C_{78}H_{98}N_8O_4{}^{4\bullet}$, 1211.69 Da.
- EPR (THF): g = 2.0053; a_N = 15.4 G; ΔH_{pp} = 1.56 G.

*1.5. tri-amino-TEMPO (**5**)*

31.7 mg of tri-imino-TEMPO (**3**) (0.03 mmol, 1 eq) were added into a flask, 4 mL of $CHCl_3$ and 2 mL of CH_3OH were added to dissolve the reagent. The flask was put into an ice bath. $NaBH_4$ (0.1 mmol, 3.66 mg) was added into the flask. Then, the reaction mixture was stirred in an ice bath overnight. After that, the reaction solution was concentrated, and DCM was added into the flask to dissolve the product and extracted with water three times. The product was obtained after the organic phase was dried (31.0 mg, 97.17%).

Characterization:

- ^1H NMR (phenylhydrazine) (250 MHz, DMSO-d_6): δ = 1.0 (d, 36H, H_a), 1.2-1.8 (m, 12H, H_b), 2.8 (s, 3H, H_c), 3.7 (s, 6H, H_d), 7.2-7.7 (m, 20H, H_e, H_f, H_g, H_h, H_i) ppm.
- IR (cm^{-1}): 3300 ν(NH), 3024 ν(CH), 2969 ν(CH), 2926 ν(CH), 2855 ν(CH), 1585, 1510, 1456, 1360, 1310, 1241, 1217, 1175, 1104, 1017, 959, 892, 842, 795, 737, 704, 681, 640, 558, 540, 509.
- MS (MALDI-TOF, positive mode): 935.1 m/z $[M+H]^+$. Calculated: $C_{60}H_{81}N_6O_3{}^{3\bullet}$, 934.35 Da.
- EPR (THF): g = 2.0057; a_N = 15.4 G; ΔH_{pp} = 1.62 G.

*1.6. tetra-amino-TEMPO (**6**).*

30.9 mg of tetra-imino-TEMPO (**4**) (0.02 mmol, 1 eq) were added into a flask, 4 mL of $CHCl_3$ and 2 mL of CH_3OH were added to dissolve the reagent. The flask was put into an ice bath. $NaBH_4$ (2.8 mg, 0.07 mmol) was added to the flask. Then, the reaction mixture was stirred in an ice bath overnight. After that, the reaction solution was concentrated, and DCM was added into the flask to dissolve the product and the organic solution extracted with water three times. The product was obtained after the organic phase was dried (28.7 mg, 95%).

Characterization:

- ^1H NMR (phenylhydrazine) (250 MHz, DMSO-d_6): δ = 1.0 (d, 48H, H_a), 1.2-1.8 (m, 16H, H_b), 2.8 (s, 4H, H_c), 3.7 (s, 8H, H_d), 7.4-7.7 (m, 26H, H_e, H_f, H_g, H_h, H_i) ppm.
- IR (cm^{-1}): 3300 ν(NH), 3017, 2970 ν(CH), 2927 ν(CH), 2849 ν(CH), 1509, 1455, 1360, 1241, 1174, 1099, 956, 853, 798, 728, 611.
- MS (MALDI-TOF, positive mode): 1220.3 m/z $[M+H]^+$. Calculated: $C_{78}H_{106}N_8O_4{}^{4\bullet}$, 1219.76 Da.
- EPR (THF): g = 2.0057; a_N = 15.4 G; ΔH_{pp} = 1.40 G.

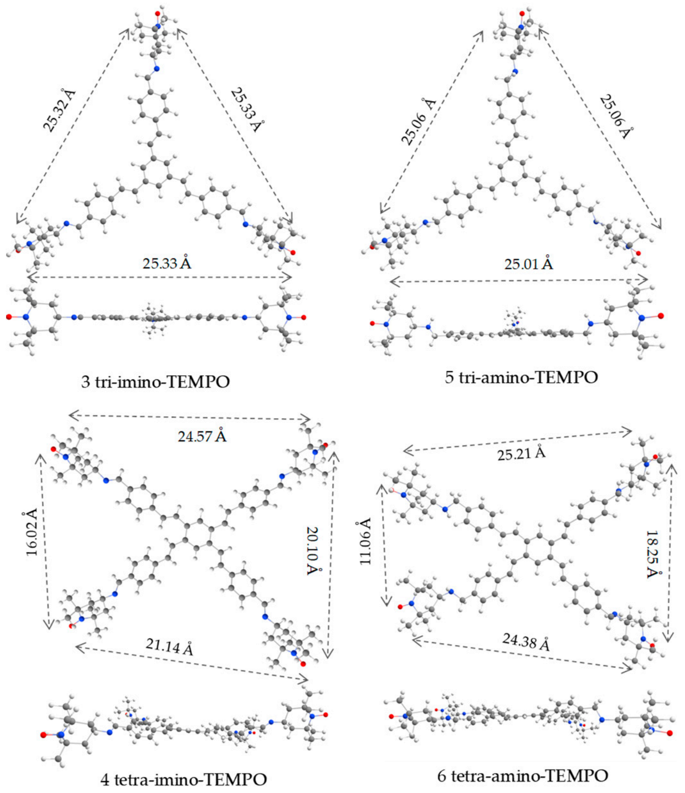

Scheme S1. Optimized geometric structures of radical dendrimers **3-6** at UB3LYP-D3(BJ)/6-31G(d) level in the gas phase.

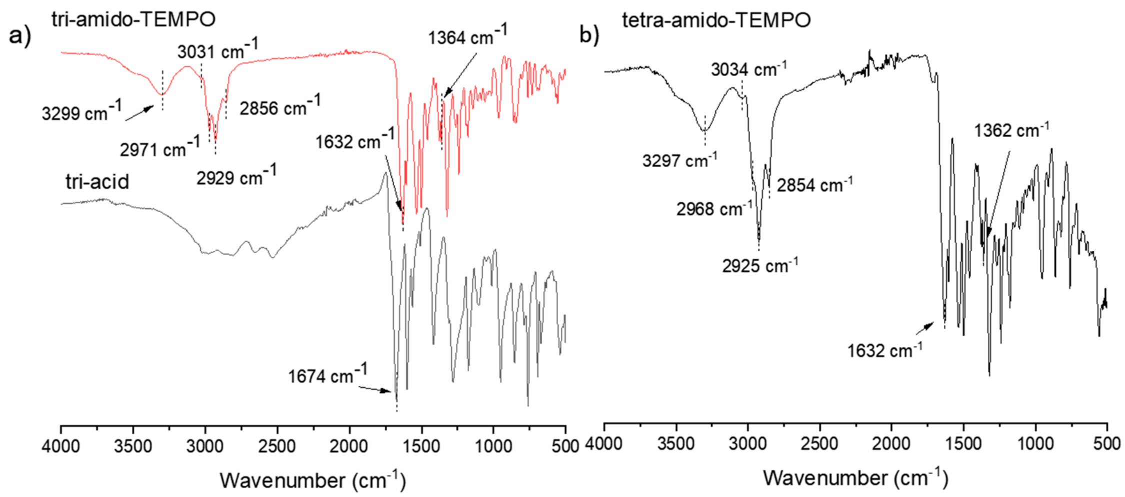

Figure S1. IR spectra of a) tri-acid **7** and tri-amido-TEMPO **1** and b) tetra-amido-TEMPO **2**.

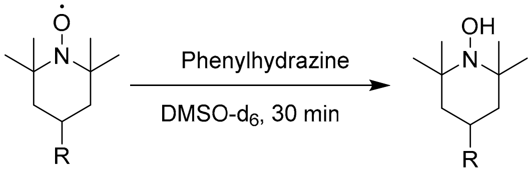

Scheme S2. Reduction of TEMPO radical to hydroxylamine with phenylhydrazine.

The UV-Vis spectrum of TEMPO radical shows two typical absorption bands: an intense band at 240 nm (Figure S2a) and a very low intensity band at ca. 450 nm (Figure S2b). By UV-Vis spectroscopy, we can characterize the radical dendrimer compounds by one hand, and on the other hand, we can also quantify the number of TEMPO radical units anchored to the dendrimers, as the low intensity n-π* transition absorption band at ca. 450 nm is known to be additive.

For this reason, the number of TEMPO units anchored to the dendrimers can be calculated, comparing the molar extinction coefficient of the ca. 450 nm absorption band of the radical dendrimers with the TEMPO free radical one, through the Lambert-Beer law. The molar extinction coefficient of the ca. 450 nm band of TEMPO radical is around 10 $M^{-1}cm^{-1}$, with small variations depending on the solvent used. For this reason, we calculated first the ε of TEMPO free radical in CH_2Cl_2, in which the radical dendrimers are soluble. We prepared five different concentrations, from 1 to 0.1 mM, and measured the maximum absorbance of the corresponding 452 nm band. The regression line obtained from the plot of A vs c gave a good regression coefficient R^2 (Figure S2c). From the slope of the regression line, we took the ε value for TEMPO in CH_2Cl_2, being 11.1 $M^{-1}cm^{-1}$.

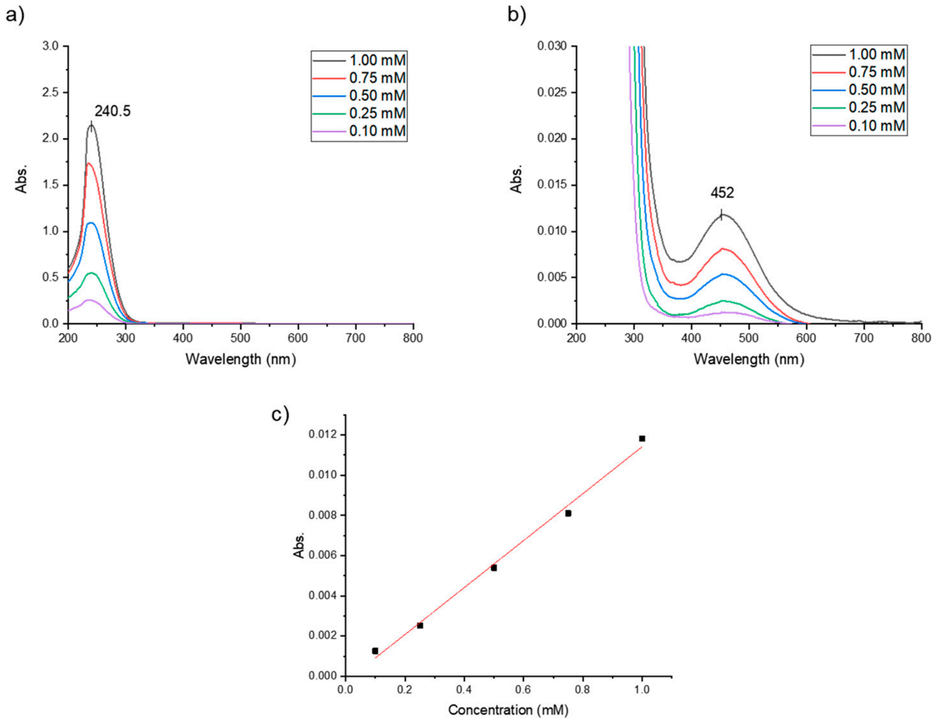

Figure S2. UV-Vis spectra of TEMPO free radical at different concentrations in DCM (1, 0.75, 0.5, 0.25, and 0.1 mM) a) the complete spectra, b) enlargement to see the 450 nm absorption band and c) absorbance versus concentration plot for the different concentrations.

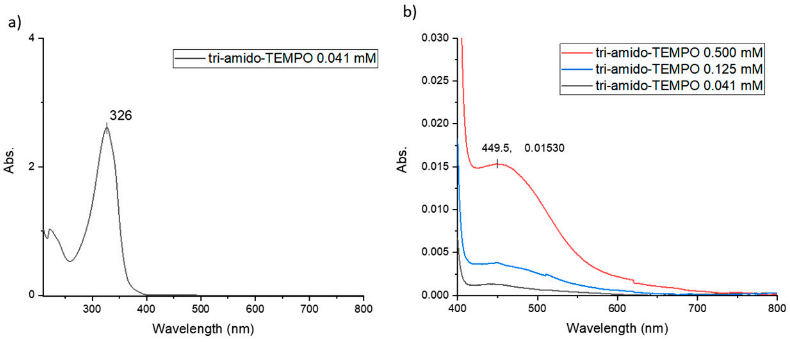

Figure S3. UV-Vis spectrum of tri-amido-TEMPO **1** in DCM, a) the complete spectrum at 0.041 mM, b) enlarged spectrum at 0.500 mM to see the 450 nm TEMPO radical absorption band.

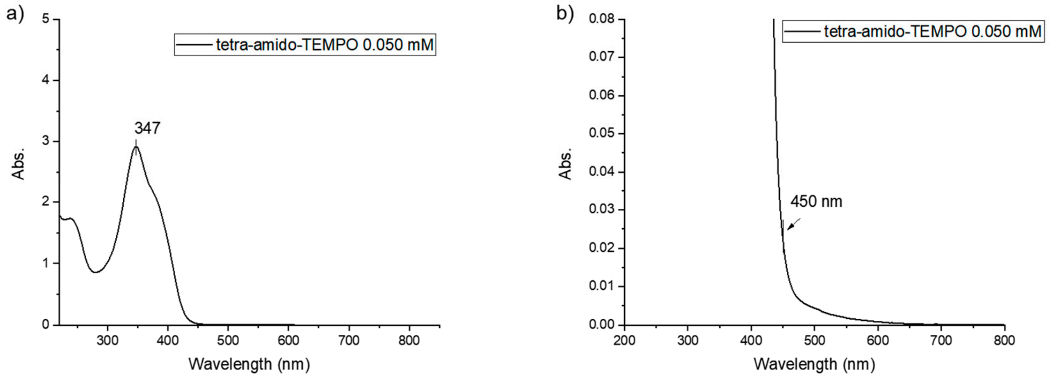

Figure S4. UV-Vis spectra of tetra-amido-TEMPO **2** in dichloromethane at 0.050 mM, a) the complete spectra, b) enlargement to see the 450 nm TEMPO radical absorption band.

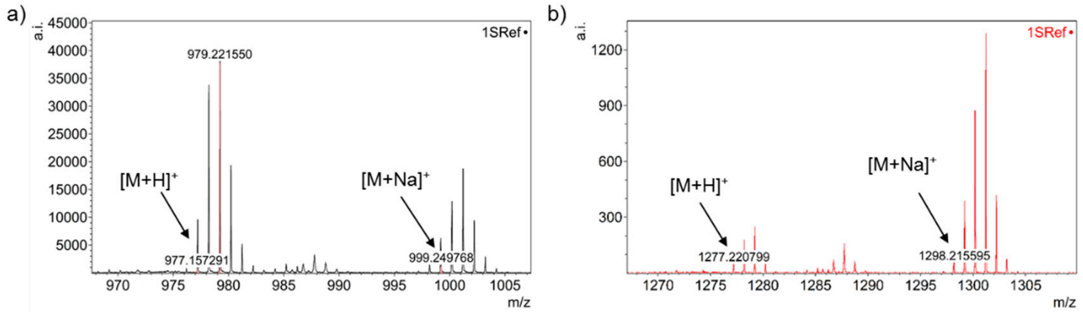

Figure S5. MALDI-TOF mass spectra of a) tri-amido-TEMPO (**1**) and b) tetra-amido-TEMPO (**2**) (matrix, positive mode).

Characterization of compounds 3-6

The IR spectra of tri- and tetra-imino-TEMPO (**3** and **4**) and tri- and tetra-amino-TEMPO (**5** and **6**) are shown in Figures S6 and S7. In the case of imino- radical dendrimers derivatives, we have followed the disappearance of the C=O stretching bands from the –CHO group at 1690 cm^{-1} of tri- and tetra-aldehyde, and the appearance of the new C=N band of the imino group at 1637 cm^{-1} for both compounds. In the case of amino- radical dendrimers derivatives, it is clearly observed that the bands at 1637 cm^{-1} of the imino (C=N) bond completely disappeared, and the new bands at 3300 cm^{-1} corresponding to the amino group formation appeared, confirming that the imino-TEMPO radical dendrimers were completely reduced to the corresponding amino derivatives. In the four compounds, we observed the -CH stretching bands of $–CH_3$ and $–CH_2$- groups of TEMPO radicals at the range ca. 2857-2972 cm^{-1} as well as the bands at 1360 cm^{-1}, assigned to the N-O$^{\bullet}$ stretching of the TEMPO radicals.

The ^{1}H NMR spectra of tri- and tetra-imino-TEMPO (**3** and **4**) and tri- and tetra-amino-TEMPO (**5** and **6**) were also obtained by using phenylhydrazine as a reducing agent, and are shown in Figures S8-S11 with the corresponding peak labeling. In the case of imino- radical dendrimers derivatives, the characteristic peaks of TEMPO protons can be found between 1.13-1.64 ppm (*a*, *b*) and 3.64 or 3.65 ppm (*c*), while the proton peaks of the dendrimer structure (*d*, *e*, *f*, *g*, *h*, *i*) can be found between 7.42 and 8.47 ppm. Importantly, the peak of aldehyde groups at 9.8 ppm disappeared in both cases **3** and **4**, which confirmed the full functionalization of both dendrimers with radicals. The peaks of the new imino groups can be found at around 8.5 ppm. In the case of amino- radical dendrimers derivatives, the peaks of the imine groups at 8.5 ppm of the imino radical dendrimers disappeared, while the new peaks at 3.7 ppm corresponding to the new formed methylene groups (**-**CH_2**-**) appeared. At the same time, the chemical shift of the methine (-CH-) groups of TEMPO changed from 3.6 ppm to 2.8 ppm, due to the close imine group changed to an amine group. Besides, in the four compounds, the number of radical units obtained by the relative integral values of ^{1}H resonances was well consistent with the theoretical values, confirming the number of anchored TEMPO radical units (3 and 4), respectively.

By UV-Vis spectroscopy, the absorption band of the radical at around 450 nm was only observed in tri-amino-TEMPO radical dendrimer (**5**) (see Figure S12) and the number of radicals could be calculated according to the extinction coefficient of such a band (30.98 $M^{-1}cm^{-1}$) which was 3 times larger than that of the free radical TEMPO. This also confirms in turn that their precursor tri-imino-TEMPO (**3**) also presented three radicals. In the other three compounds (**3**, **4**

and **6**) the characteristic TEMPO absorption band was overlapped by the OSB dendrimers band (Figure S13 of the Supporting Information).

The MALDI-TOF mass spectra of tri- and tetra-imino-TEMPO (**3** and **4**) and tri- and tetra-amino-TEMPO (**5** and **6**) presented the corresponding molecular ion peak $[M+H]^+$ at m/z = 929.2 and 1213.2 for **3** and **4**, respectively, and at 935.1 and 1220.3 m/z, for **5** and **6**, respectively (Figure S14 and S15), in agreement with the theoretical molecular mass (928.30, 1211.69, 934.35 and 1219.76 Da respectively), confirming the formation of the radical dendrimers.

Tri- and tetra-imino-TEMPO (**3** and **4**) radical dendrimers were also characterized by SEC (Figure S16). In both cases, their chromatograms showed a single and narrow size distribution band and, as expected, the retention time of tri-imino-TEMPO **3** was slightly larger than tetra-imino-TEMPO **4**. In the case of tri- and tetra-amino-TEMPO (**5** and **6**) we obtained peaks of very low intensity. However, even we had more error the retention time was: 17.04 and 16.96 min for tri- and tetra-amino-TEMPO (**5** and **6**), respectively.

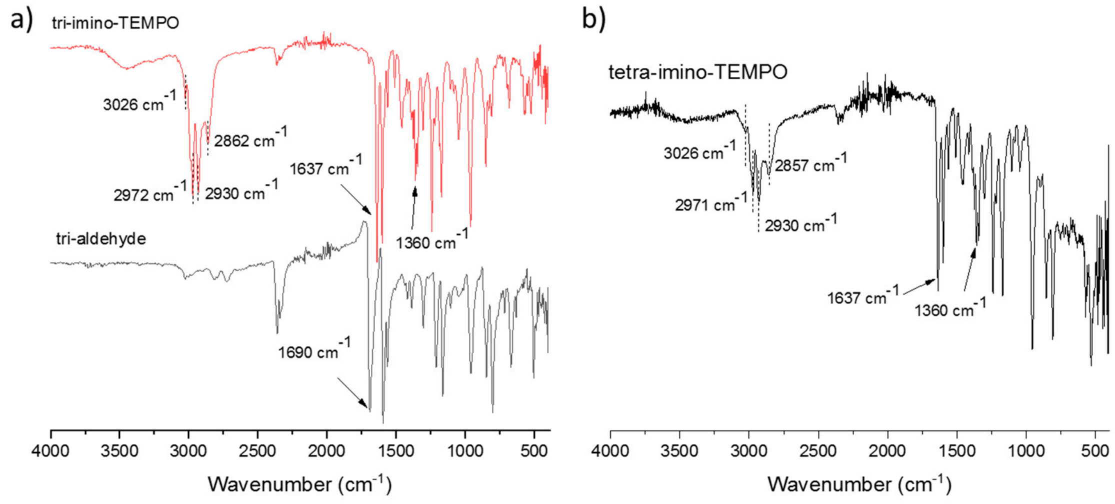

Figure S6. IR spectra of a) tri-aldehyde **9** and tri-imino-TEMPO **3**, and b) tetra-imino-TEMPO **4**.

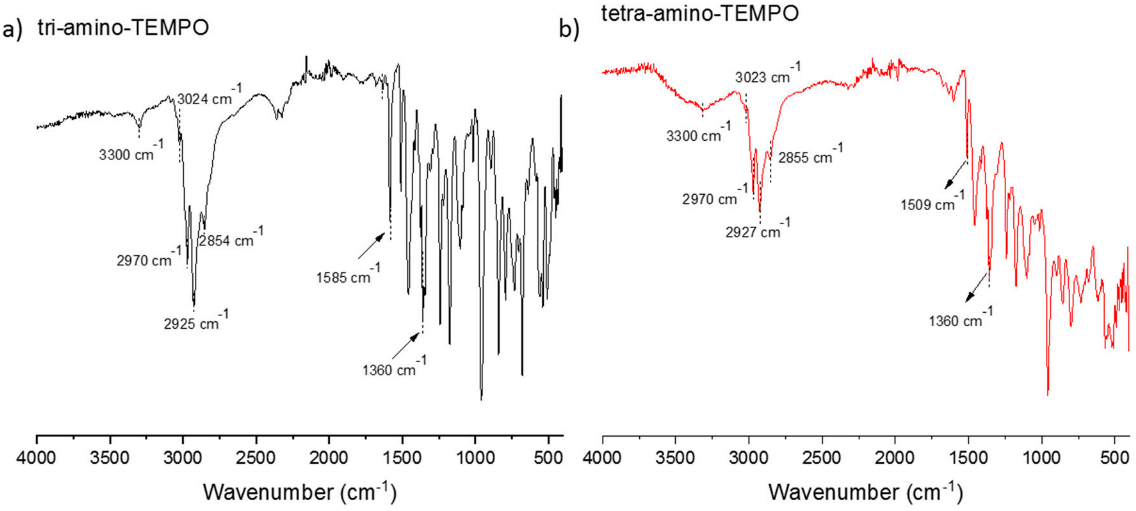

Figure S7. IR spectra of a) tri-amino-TEMPO **5** and b) tetra-amino-TEMPO **6** radical dendrimers.

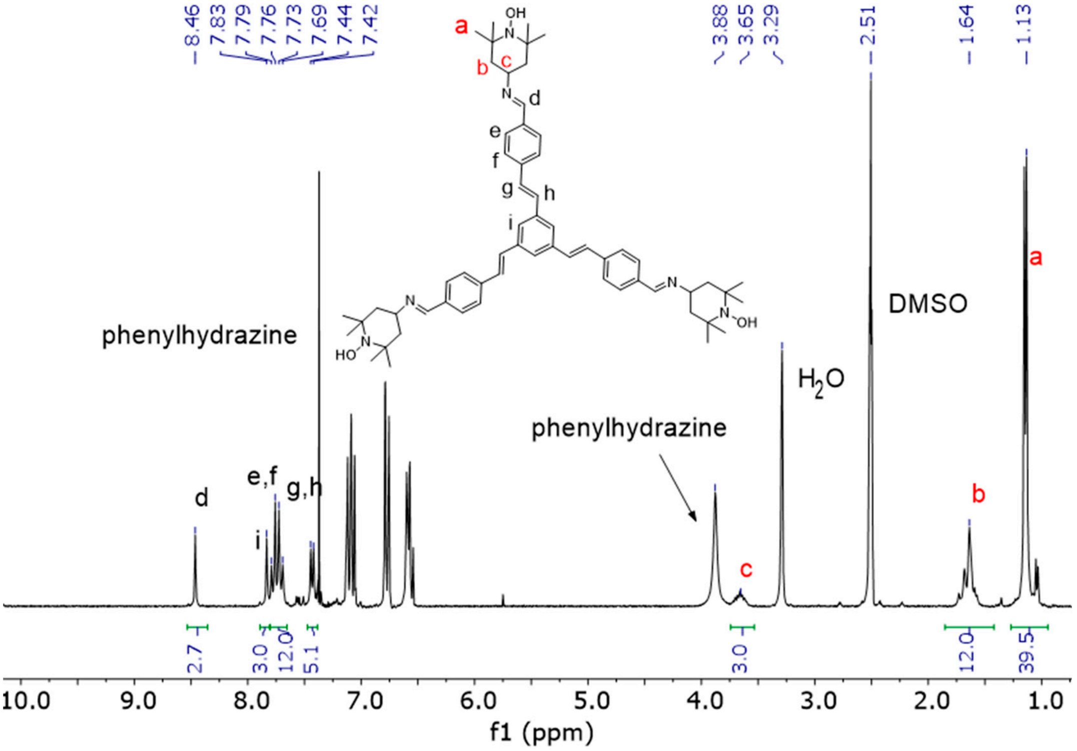

Figure S8. ^{1}H NMR spectrum of tri-imino-TEMPO **3** after being reduced with phenylhydrazine (DMSO-d_6, 250 MHz).

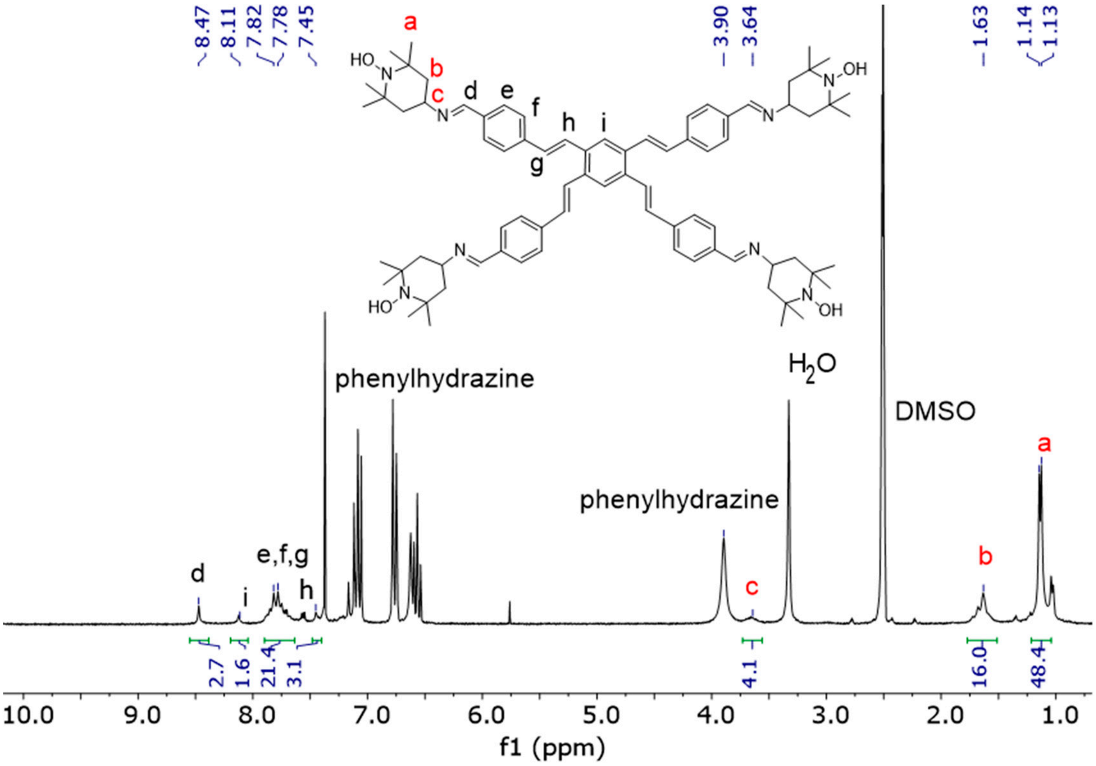

Figure S9. ^{1}H NMR spectrum of tetra-imino-TEMPO **4** after being reduced with phenylhydrazine (DMSO-d_6, 250 MHz).

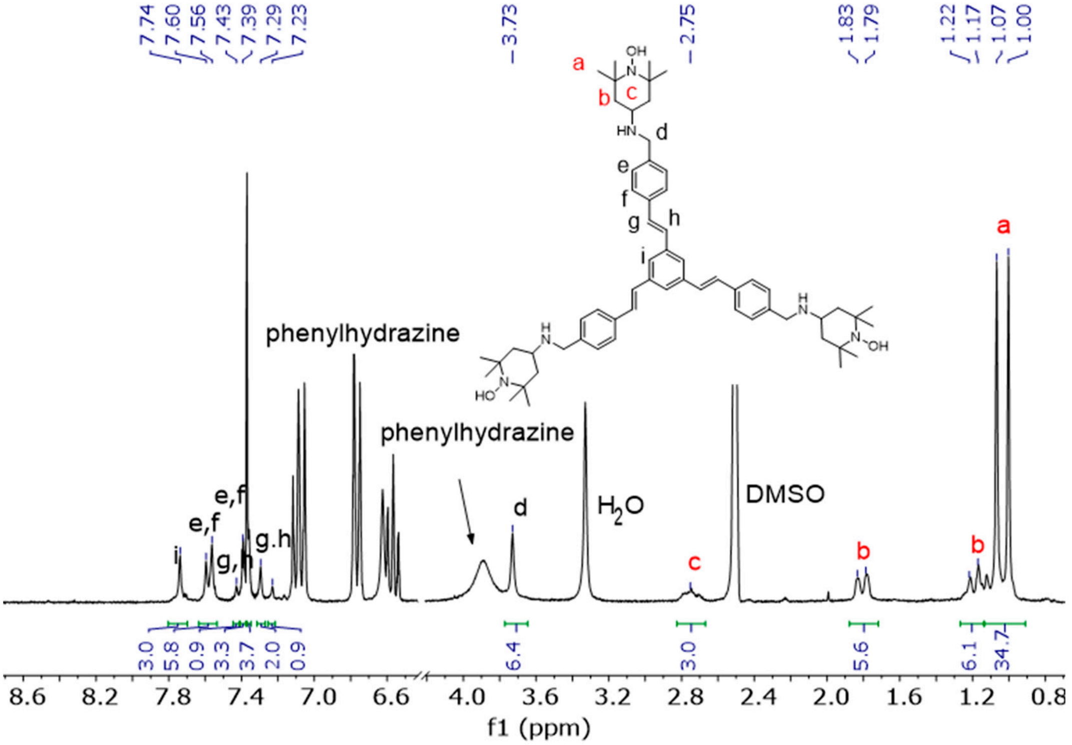

Figure S10. ^{1}H NMR spectrum of tri-imino-TEMPO **5** after being reduced with phenylhydrazine (DMSO-d$_6$, 250 MHz).

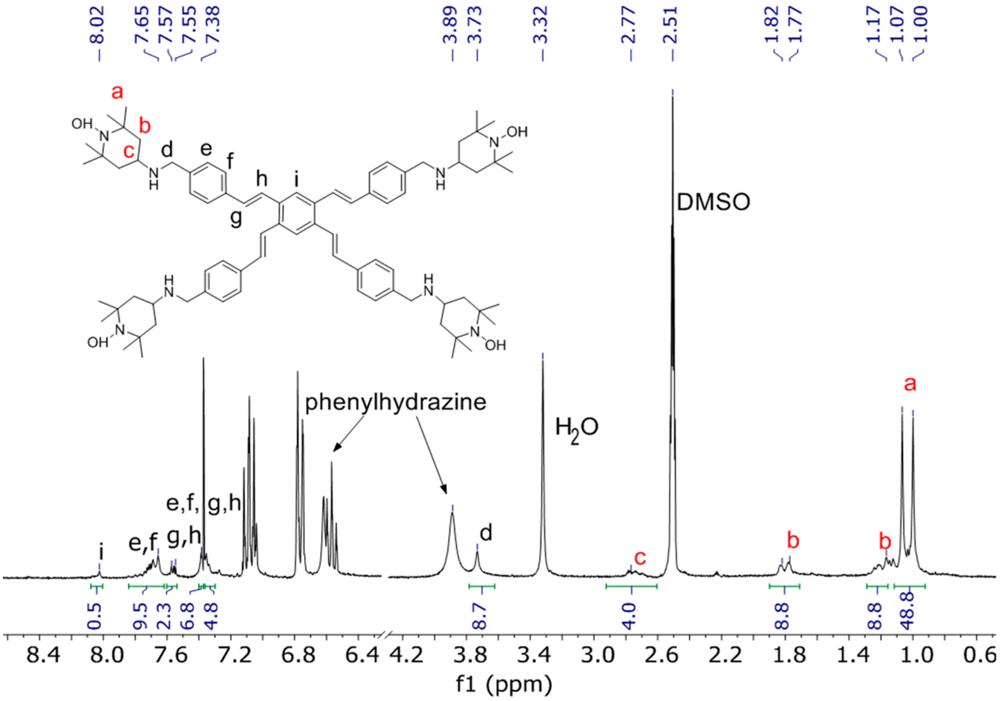

Figure S11. ^{1}H NMR spectrum of tri-imino-TEMPO **6** after being reduced with phenylhydrazine (DMSO-d$_6$, 250 MHz).

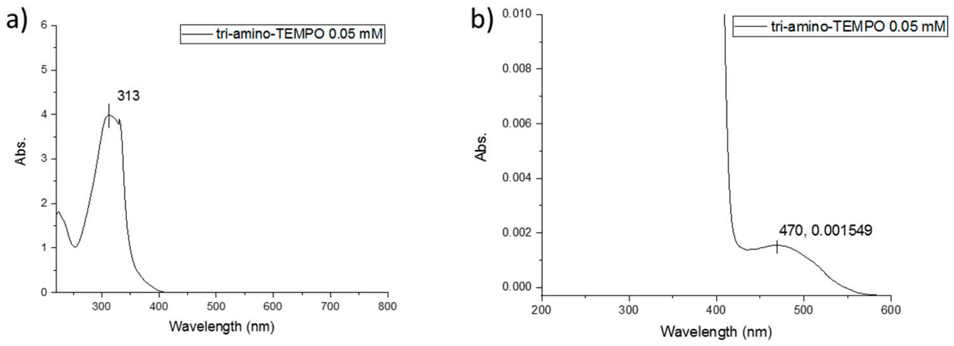

Figure S12. UV-Vis spectra of tri-imino-TEMPO **5** in dichloromethane at 0.05 mM a) the complete spectrum, b) enlargement to see the TEMPO radical absorption band.

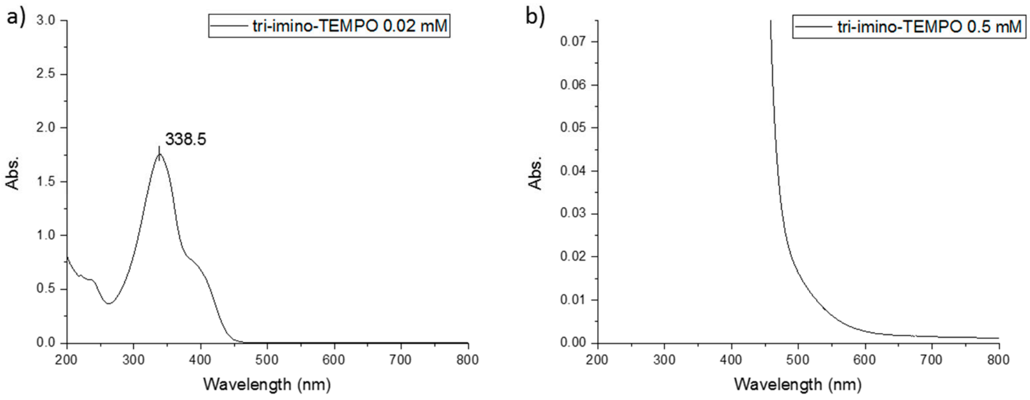

Figure S13. UV-Vis spectra of tri-imino-TEMPO **3** in dichloromethane, a) the complete spectrum at 0.02 mM, b) enlargement to see the 450 nm TEMPO radical absorption band at 0.5 mM.

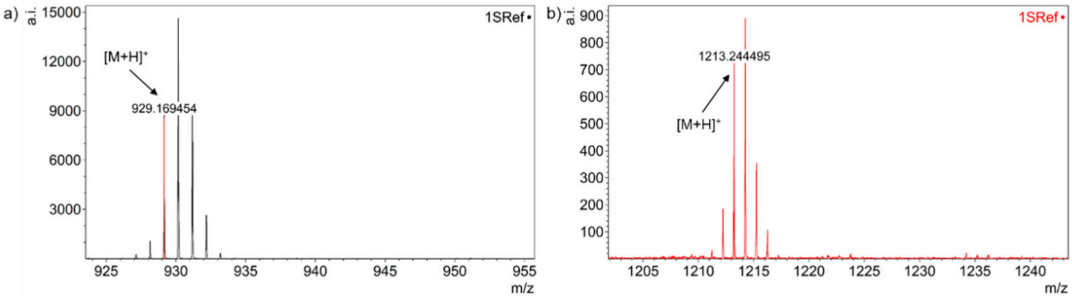

Figure S14. MALDI-TOF mass spectra of a) tri-imino-TEMPO **3** and b) tetra-imino-TEMPO **4** (matrix, positive mode).

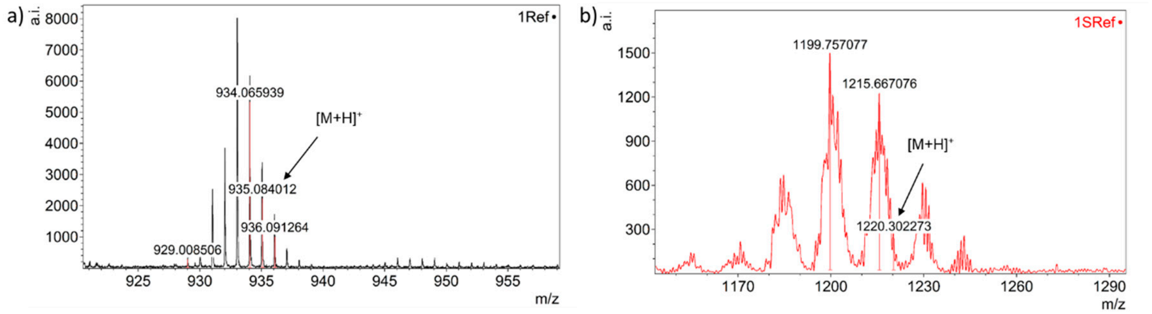

Figure S15. MALDI-TOF mass spectra of a) tri-amino-TEMPO **5**, and b) tetra-amino-TEMPO **6** (matrix, positive mode).

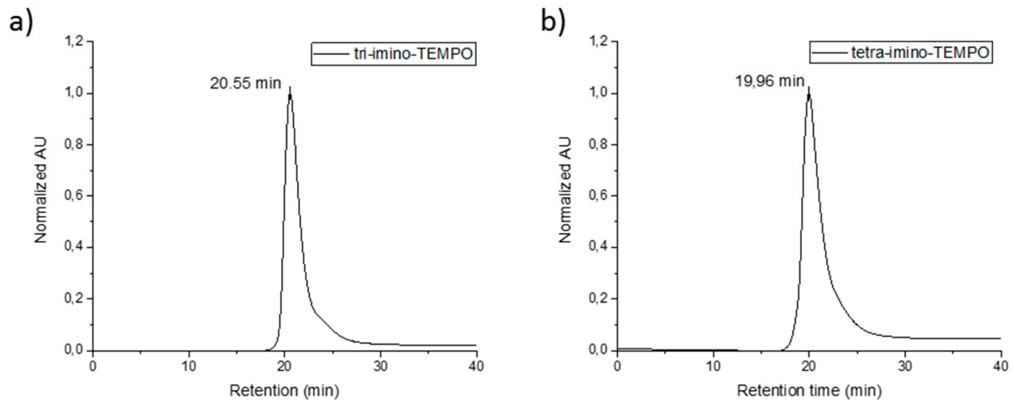

Figure S16. SEC chromatograms of a) tri-imino-TEMPO **3** and b) tetra-imino-TEMPO **4** radical dendrimers.

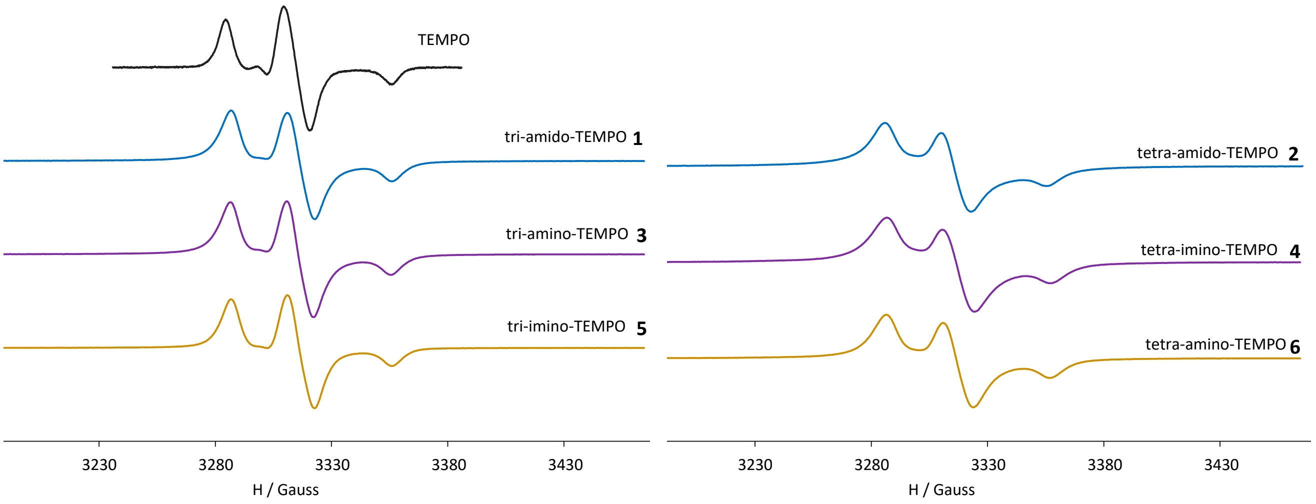

Figure S17. EPR spectra of TEMPO, tri-amido-TEMPO **1**, tetra-amido-TEMPO **2**, tri-imino-TEMPO **3**, tetra-imino-TEMPO **4**, tri-amino-TEMPO **5** and tetra-amino-TEMPO **6** radical dendrimers in THF at 120 K.

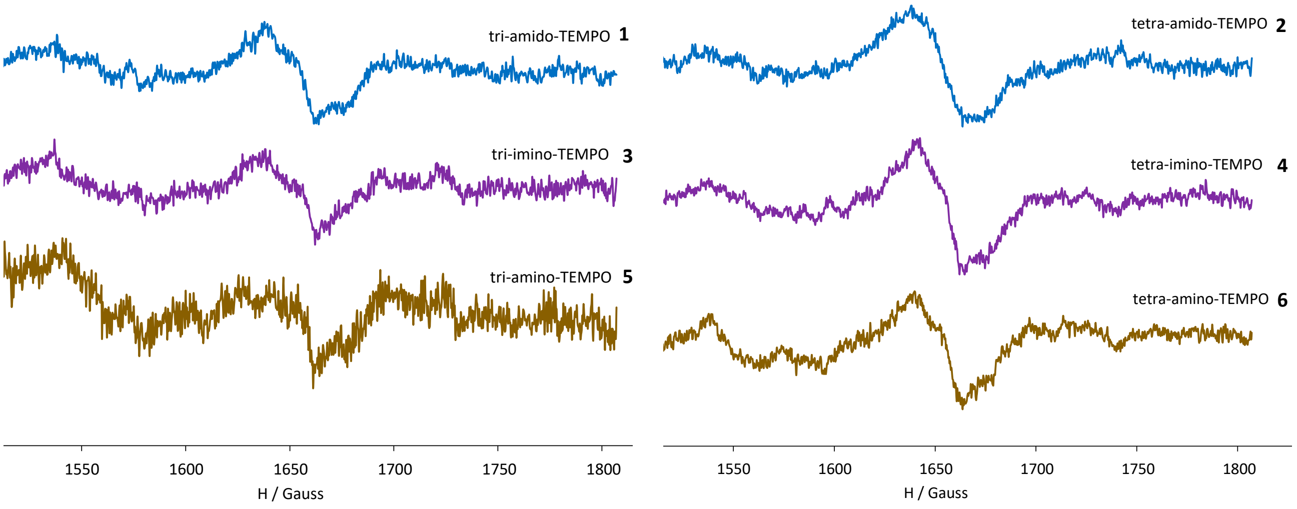

Figure S18. EPR spectra of $|\Delta m_S| = 2$ transition at half-field of tri-amido-TEMPO **1**, tetra-amido-TEMPO **2**, tri-imino-TEMPO **3**, tetra-imino-TEMPO **4**, tri-amino-TEMPO **5** and tetra-amino-TEMPO **6** radical dendrimers in THF at 120 K.

2. Measurement of quantum yield

The fluorescence process is schematized in the following Jablonski diagram:

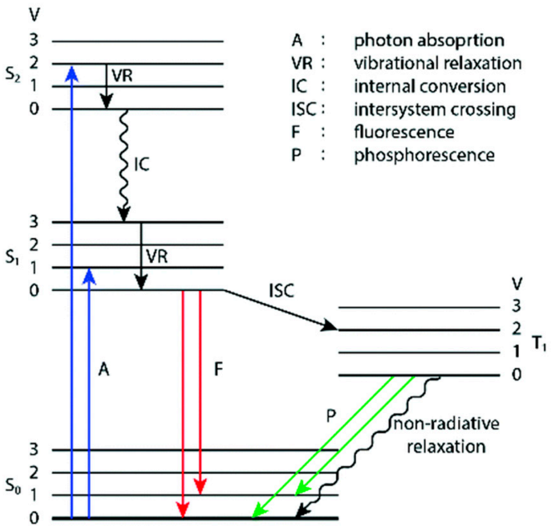

The quantum yield has been calculated in the following way:

$$QY = \frac{\mathrm{F}_S \times A_r \times R_S^2 \times QY_r}{\mathrm{F}_r \times A_S \times R_r^2}$$

where:

Fs: Fluorescence peak area of the sample

Ar: Absorbance of the reference

Rs: Refraction index of the solvent used to dissolve the sample

QYr: QY of the reference (quinine sulfate)

Fr: Fluorescence peak area of the reference

As: Absorbance of the sample

Rr: Refraction index of the solvent used to dissolve the reference

a)

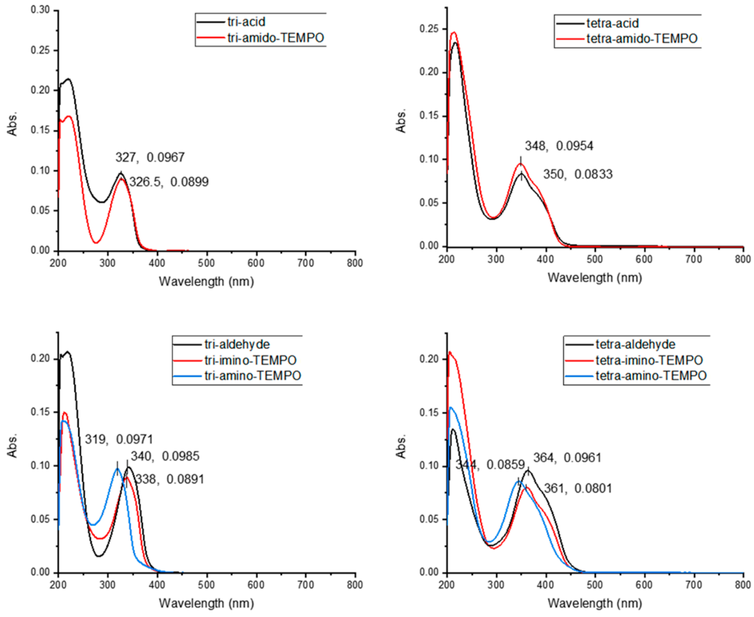

b)

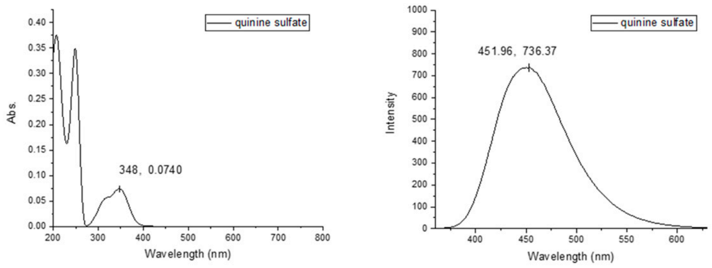

Figure S19. a) UV-Vis spectra of amido-, imino- and amino- radical dendrimers derivatives **1**-**6** in THF. b) left) UV-Vis spectrum and right) fluorescence emission spectrum of the standard quinine sulfate.

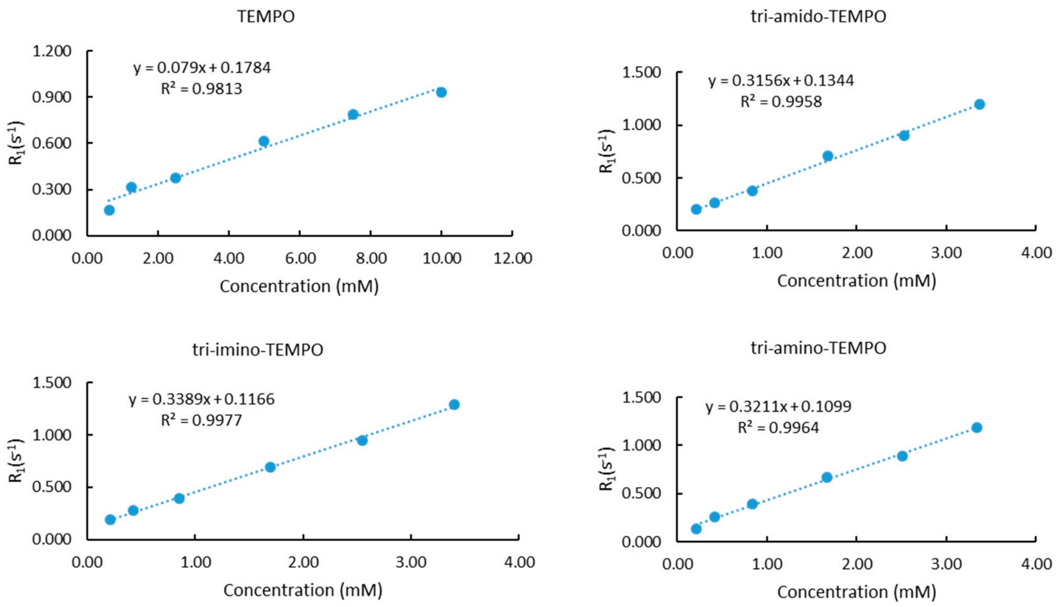

Figure S20. Plots of R_1 (1/ T_1) versus concentration for different concentrations of free TEMPO, tri-amido-TEMPO **1**, tri-imino-TEMPO **3** and tri-imino-TEMPO **5** in dichloromethane.

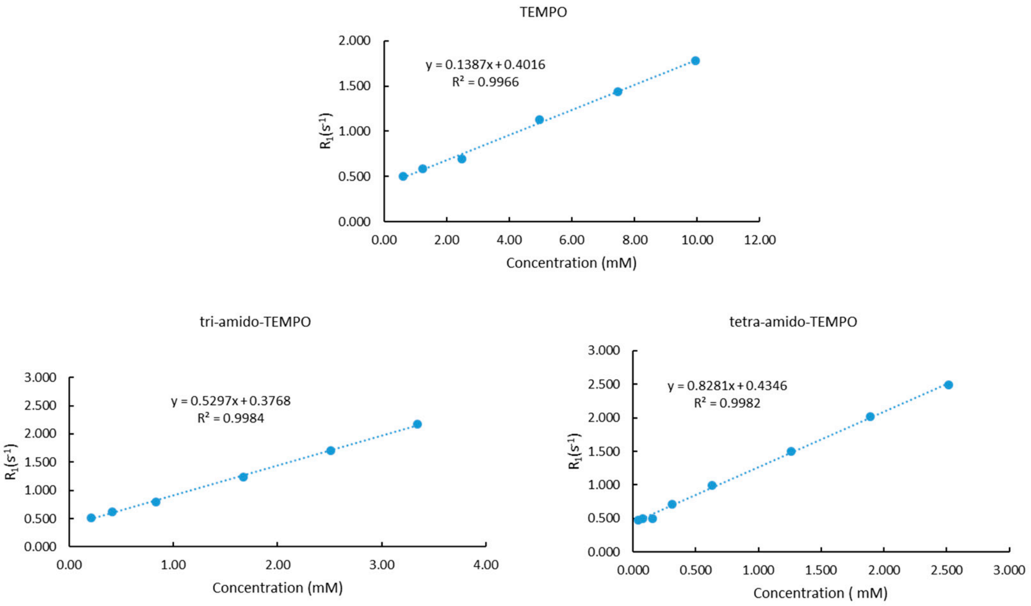

Figure S21. Plots of R_1 (1/ T_1) versus concentration for different concentrations of TEMPO, tri-amido-TEMPO **1** and tetra-amino-TEMPO **2** in DMSO.
